# *luxS* contributes to intramacrophage survival of *Streptococcus agalactiae* by positively affecting the expression of *fruRKI* operon

**DOI:** 10.1186/s13567-023-01210-9

**Published:** 2023-09-27

**Authors:** Qing Cao, Yuhao Dong, Changming Guo, Shuting Ji, Meng Nie, Guangjin Liu, Xihe Wan, Chengping Lu, Yongjie Liu

**Affiliations:** 1 Joint International Research Laboratory of Animal Health and Food Safety, College of Veterinary Medicine, Nanjing Agricultural University, Nanjing, Jiangsu China; 2 Jiangsu Agri-Animal Husbandry Vocational College, Taizhou, Jiangsu China; 3 Institute of Oceanology and Marine Fisheries, Nantong, China

**Keywords:** *Streptococcus agalactiae*, *luxS*, *fruRKI* operon, CcpA, immune evasion

## Abstract

The LuxS quorum sensing system is a widespread system employed by many bacteria for cell-to-cell communication. The *luxS* gene has been demonstrated to play a crucial role in intramacrophage survival of piscine *Streptococcus agalactiae*, but the underlying mechanism remains largely unknown. In this study, transcriptome analysis, followed by the *luxS* gene deletion and subsequent functional studies, confirmed that impaired bacterial survival inside macrophages due to the inactivation of *luxS* was associated with reduced transcription of the *fruRKI* operon, encoding the fructose-specific phosphotransferase system. Further, *luxS* was determined not to enhance the transcription of *fruRKI* operon by binding its promoter, but to upregulate the expression of this operon via affecting the binding ability of catabolite control protein A (CcpA) to the catabolite responsive element (*cre*) in the promoter of *fruRKI*. Collectively, our study identifies a novel and previously unappreciated role for *luxS* in bacterial intracellular survival, which may give a more thorough understanding of the immune evasion mechanism in *S. agalactiae*.

**Supplementary Information:**

The online version contains supplementary material available at 10.1186/s13567-023-01210-9.

## Introduction

*Streptococcus agalactiae*, also known as group B *streptococcus* (GBS), is an important pathogen of both humans and animals [1]. This bacterium was first isolated from cow milk with mastitis, and subsequently, it was found to be a major etiologic agent of neonatal sepsis and meningitis [2]. Although several mortality events associated with bacterial infection in fish were reported in the 1980s, not much attention was paid at the outset because of the limited epidemic area. However, since 2009, large-scale streptococcal outbreaks caused by this bacterium continuously occurred in tilapia farms with high mortality and brought a deleterious impact on aquaculture industry worldwide [3]. Pathological phenotypes of *S. agalactiae* infection are mainly septicemia and meningoencephalitis in farmed tilapia [4]. Most notably, episodes of bacteremia and meningitis in humans were recently reported to be associated with consumption of raw fish infected with GBS sequence type (ST) 283 in Singapore [5–7] and Hong Kong (official figures).

Pathogenic mechanisms of meningoencephalitis caused by bacteria have been extensively researched in recent years. The mechanism by which pathogens target the brain and cross the blood–brain barrier (BBB) in the early phase of infection is dependent on successful evasion of the host innate immune system. Phagocytes play central roles in the innate immune response, and bacterial survival within phagocytes may contribute to dissemination of the pathogen within its host. Pathogens have developed diverse strategies to survive within phagocytes and even to take advantage of the intracellular environment. For example, *Staphylococcus aureus* is able to perturb the acquisition of lysosomal hydrolases, e.g., cathepsin D and β-glucuronidase, in macrophages, thereby preventing its degradation in the phagolysosome [8]. In *Streptococcus pneumoniae*, pneumolysin (Ply), a member of the thiol-activated cytolysin family of toxins, could inhibit the initial macrophage inflammatory response and improve bacterial immune evasion [9]. In *Streptococcus pyogenes*, a carbohydrate metabolism-related operon called *fruRBA* was found to be critical for the survival of this bacterium in neutrophils [10]. *S. agalactiae* has been known to utilize multiple virulence factors to survive inside host phagocytes. To defend itself against oxidative stress and reduce ROS production inside macrophages, this bacterium expresses an NADH-dependent peroxidase [11]. Also, hyaluronidase has a positive influence on the intracellular survival of *S. agalactiae* by inhibiting the secretion of proinflammatory cytokines [12]. Our previous study indicated that S-ribosylhomocysteine (SRH) lyase (LuxS) contributes to the intracellular survival of *S. agalactiae* within macrophages [13]. But paradoxically, the *luxS* inactivation has been reported to enhance the intracellular survival of *S.* *pyogenes* [14] or *S. aureus* [15].

As a homodimer, LuxS can formally catalyze the non-redox cleavage of bonded sulfides in S-rybosylhomocysteine (SRH) to produce L-homocysteine and 4,5-dihydroxy-2,3-pentanedione (DPD), which spontaneously cyclizes to active the autoinducer-2 molecule (AI-2) [16]. It is well-known that AI-2 serves as a universal signaling molecule in quorum sensing (QS) which mediates both intra- and interspecific communication [17]. Many studies have demonstrated that LuxS can regulate bacterial physiological processes through AI-2, such as biofilm formation [18], swimming motility [19] and antibiotic resistance [20]. However, our study has demonstrated that LuxS contributes to intracellular survival of *S. agalactiae* independent of the effect of AI-2 [13]. Knowledge about the contribution of LuxS/AI-2 to bacterial intracellular survival is rather lacking.

In this study, we explored the mechanism by which LuxS is responsible for the intracellular survival of *S. agalactiae* GD201008-001. We found that inactivation of *luxS* caused highly downregulated expression of the *fruRKI* genes as an operon. Interestingly, this regulation effect of *luxS* is associated with the impact on the binding ability of catabolite control protein A (CcpA) to the *fruRKI* promoter. The novel function of *luxS* identified in this study will broaden our understanding of the pathogenesis of *S. agalactiae*.

## Materials and methods

### Bacterial strains, cell lines and culture conditions

*Streptococcus* *agalactiae* GD201008-001 was isolated in 2010 from tilapia with meningoencephalitis in Guangdong Province, China [21]. GD201008-001 wild-type strain (WT) and its derived *luxS* mutant strain (Δ*luxS*) and the *luxS* complemented strain (CΔ*luxS*) [13] were maintained in Todd-Hewitt broth (THB) or in chemically defined medium (CDM) [22]. *Escherichia coli* was cultured in Luria–Bertani (LB) medium. For plasmids screening required, media were supplemented with antibiotics using the concentration below: 100 μg/mL spectinomycin (Sp, Sigma, St. Louis, MO, USA), 10 μg/mL erythromycin (Em, Sigma), 100 μg/mL kanamycin (Km, Sigma) or 100 μg/mL ampicillin (Ap, Sigma). The details of bacterial strains and plasmids are listed in Additional file 1. Macrophage cell line RAW 264.7 were maintained in DMEM (Gibco, Grand Island, NY, USA) supplemented with 10% fetal bovine serum (FBS, Gibco).

### DNA methods and construction of mutant strains

PCR for cloning and generating fragment fusions was performed using 2 × Phanta Max Master Mix (Vazyme, Nanjing, China) and diagnostic assays were performed using Green Taq Mix (Vazyme) following the manufacturer’s protocol. DNA fragments and plasmids digested with enzymes were gel purified from agarose using the Gel Extraction Kit (Omega, Beijing, China). All DNA sequencing was done by Genewiz, Inc (Suzhou, China).

The whole *fruRKI* operon was knocked out using the suicide plasmid pSET4S [23] and performed according to the method described previously with some modulations [24]. All primers used in this study are listed in Additional file 2. For construction of the Δ*fruRKI* mutant, DNA fragments flanking the *fruRKI* operon were amplified using the primer pairs *fruRKI* 1/2 (before the start codon of *fruR*) and *fruRKI* 3/4 (after the stop codon of *fruI*), fused together using the primer pair *fruRKI* 1/4, and then cloned into the pSET4S. The recombinant plasmid was transformed into the chemically competent *E. coli* DH5α. After sequencing, the recombinant plasmid was transformed into the *S. agalactiae* GD201008-001 competent cells by electroporation. After electroporation, the strain containing plasmid was sub-cultured twice daily at 28 °C for five days and then THB medium with Sp was used to check for plasmid loss after the successful double-crossover recombination between plasmid and genome. The Δ*fruRKI* mutant by double-crossover recombination was confirmed by amplifying the *fruRKI* locus using primers *fruRKI*-F and *fruRKI*-R followed by DNA sequencing.

To construct the *fruRKI* complementary stain (CΔ*fruRKI*), the whole operon with the flanking fragments was amplified and cloned into the pSET4S. The recombinant plasmid was transformed into the Δ*fruRKI* strain. The resultant strain was cultured on Sp-containing THB agar medium, and positive clones were verified by PCR followed by DNA sequencing. The *cre* site mutation strains were constructed following a similar procedure as above, except that the fragment containing the point mutation was synthesized and cloned into the pSET4S. The mutation sites of the *cre* were chosen based on the conserved critical nucleotides identified by sequence alignment. In this study, five strains with the *cre* site mutation were generated, i.e., WT/Δ*luxS*-G1 (the conserved nucleotides of the *cre* region were replaced in the *fruRKI* promoter of the WT or the Δ*luxS* strain), and WT/Δ*luxS*-G2 (control strains, non-conserved nucleotides of the *cre* were replaced in the *fruRKI* promoter).

The shuttle vector pSET2 [25] that drives *fruI*, *fruK* or *fruR*, respectively, were constructed to complement the loss of each gene function of *fruRKI* operon in the Δ*luxS* mutant strain. For Δ*luxS*::*fruI* strain, the promoter region of *fruRKI* (amplified by the primer pair C*fruI*1/2) and the *fruI* coding sequence (amplified by the primer pair C*fruI*3/4) was fused together using the primer pair C*fruI*1/4 and then cloned into pSET2. The recombinant plasmid was transformed into the Δ*luxS* competent cells by electroporation and the Δ*luxS*::*fruI* strain was selected by THB medium with Sp.

To construct a promoter reporter strain, the 129 bp-DNA fragment containing the promoter region of *fruRKI* operon was amplified using the primers P*fruRKI*-lacZ-F/P*fruRKI*-lacZ-R and cloned in front of a promoterless *β*-galactosidase gene in a shuttle vector, pTCV-lac [26]. The resultant strain was named P*fruRKI*-lacZ. For point mutations on pTCV-lac plasmids, two DNA fragments with 5’ homology arms containing mutation sites were amplified and fused, and cloned into pTCV-lac. All resulting plasmids were sequenced and then transformed into the wild-type *S. agalactiae* GD201008-001 and its derivative mutants by electroporation and named PM*fruRKI*-lacZ, T1P*fruRKI*-lacZ and T2P*fruRKI*-lacZ.

### Bacterial growth in carbon defined medium

For the carbohydrate metabolic assay, *S. agalactiae* strains to be tested were cultured in THB medium at 37 °C with 180 rpm until to reach the stationary phase and then adjusted to the optical density at 600 nm (OD_600_) of 0.8. The adjusted suspensions were inoculated into 100 mL of CDM supplemented with 0.1% (w/v) of either glucose or fructose (Sigma) as a sole carbon source at a ratio of 1:100 and incubated under the same conditions for 1 day. The cell densities at OD_600_ were measured every 2 h. The experiment was repeated three times independently.

### Transcriptome analysis

Bacteria were cultured in THB medium at 37 °C. After reaching an OD_600_ of 0.8, bacterial RNA was extracted by an RNAqueous kit (Thermo Fisher Scientific, San Jose, CA, USA). Before the RNA library assembly, ribosomal RNA was removed using Ribo-Zero Magnetic Kit (Illumina, San Diego, CA, USA). Libraries construction and transcriptome sequencing were conducted by OE Biotech (Shanghai, China). A GO enrichment analysis was conducted by the OECloud tools based on GO Database. To perform RNA-Seq, the *S. agalactiae* strains were purified and divided into three for triplicate repeats. After that, total RNA of each repeat was extracted and sent for RNA-Seq. All samples were analyzed in one sequencing run.

### Real-time quantitative PCR (RT-qPCR)

The RT-qPCR was carried out to measure the transcription levels of target genes using the primers listed in Additional file 3. The total RNA was extracted using the Total RNA kit (Omega, Norcross, GA, USA) and then reverse transcribed into cDNA by Hiscript II QRT Supermix (Vazyme). The mRNA levels of target genes were measured by RT-qPCR according to the protocol of One Step RT-qPCR SYBR Green kit (Vazyme). The *recA* gene was used as an internal control. The fold-changes of mRNA levels were calculated using the comparative cycle threshold (2^−ΔΔCT^) method [27]. The experiment was repeated three times independently.

### *S. agalactiae* intracellular survival and phagocytosis assay

RAW264.7 cells were cultured in 24-well plates at a density of 2 × 10^5^ cells/well with 10% FBS added DMEM at 37 °C with 5% CO_2_ for 20 h, until to 85–90% confluence. *S. agalactiae* strains were cultured in THB medium overnight at 37 °C. Then bacteria were washed three times in PBS and adjusted to 4 × 10^6^ bacterial cells/mL using DMEM. The cells were washed and then inoculated with *S. agalactiae* at a multiplicity of infection (MOI) of 1:1 for 1 h. To eliminate residual adherent bacteria, the cells were washed five times and then added 10% FBS-DMEM containing 1% penicillin G and incubated for 1 h.

To measure the phagocytotic rate, infected cell samples were taken 1 h after antibiotic treatment and subjected to lysis. Ten-fold serial dilution of lysates were made with PBS and then cultured on THB agar to give a bacterial count of colony forming unit (CFU). The percentage of phagocytosis was calculated based on the CFU of intracellular bacteria relative to the total CFU of bacteria added in the cell monolayers. To measure the survival rate of intracellular bacteria, the cell sampling period was started after 1 h antibiotic treatment (time point 0) and samples were taken every 4 h during 12 h period. The final timepoint of sampling was 24 h after time point 0. Infected cells were treated as above to measure the CFU of intracellular bacteria. The relative survival rate was calculated as follows: (CFU at a specific time point/CFU at time point 0) × 100. The assays described above were repeated three times with three independent replicates.

### Electrophoretic mobility shift assay (EMSA)

The LuxS and CcpA proteins were expressed and purified as previously described [13]. The ribosylhomocysteinase activity of LuxS has been confirmed by the ability to synthesize AI-2 in vitro [13]. The primers used to amplify the *ccpA* gene are listed in Additional file 2. The DNA fragments used in EMSAs were amplified by primer pairs PM*fruRKI*-F/PM*fruRKI*-R and PM*luxS*-F/PM*luxS*-R. A 70 bp DNA fragment served as the negative control. The purified protein (0.2–1.0 μM) was incubated with the DNA fragment (25 nM) in binding buffer [20 mM Tris–HCl (pH = 7.5), 30 mM KCl, 1 mM DTT, 1 mM EDTA (pH = 7.5), 10% (v/v) glycerol)] in a final volume of 20 μL for 30 min at 37 °C. Samples were loaded on a 10% polyacrylamide gel and electrophoresed in 0.5 × TBE (44.5 mM Tris, 44.5 mM boric acid, 1 mM EDTA, pH = 8.0) under 120 V with the ice bath for 1 h. The gel was stained in Gold nucleic acid staining solution for 10 min, and then watched and recorded under the UV Trans illumination by Gel Doc XR (Bio-Rad, CA, USA). The experiment was repeated three times independently.

### β-galactosidase assay

*Β*-galactosidase assay was performed as previously described [26]. *S. agalactiae* strains were cultured in THB medium for 12 h and then cultured in fresh THB to an OD_600_ of 0.8. Bacteria were incubated on ice for 20 min and washed three times in *β*-mercaptoethanol (BME) free Z buffer and adjusted to an OD_600_ of 1.0. The diluted cells were permeabilized by treatment with 0.05 M BME, 0.5% toluene and 4.5% ethanol for 5 min at 30 °C. The substrate o-nitrophenyl-*β*-D-galactoside (ONPG, 4 mg/mL) was added to start reaction until sufficient yellow color has developed. The reaction time was recorded. The activity of β-galactosidase was calculated by the formula: (10^3^) × (OD_420_−1.75 × OD_550_)/(reaction time × volume of culture × OD_600_). The experiment was repeated three times independently.

### Statistical analyses

Data were presented as the mean ± standard deviations (SD). GraphPad Prism version 8.0.1 was used for the statistical analysis and graph preparation. All statistical analyses were performed using unpaired two-tailed Student’s *t* test. Comparisons with *P* ≤ 0.05 were accepted as statistically significant.

## Results

### A number of differentially expressed genes (DEGs) were identified in the luxS deficiency mutant of *S. agalactiae*

**Fig. 1:**
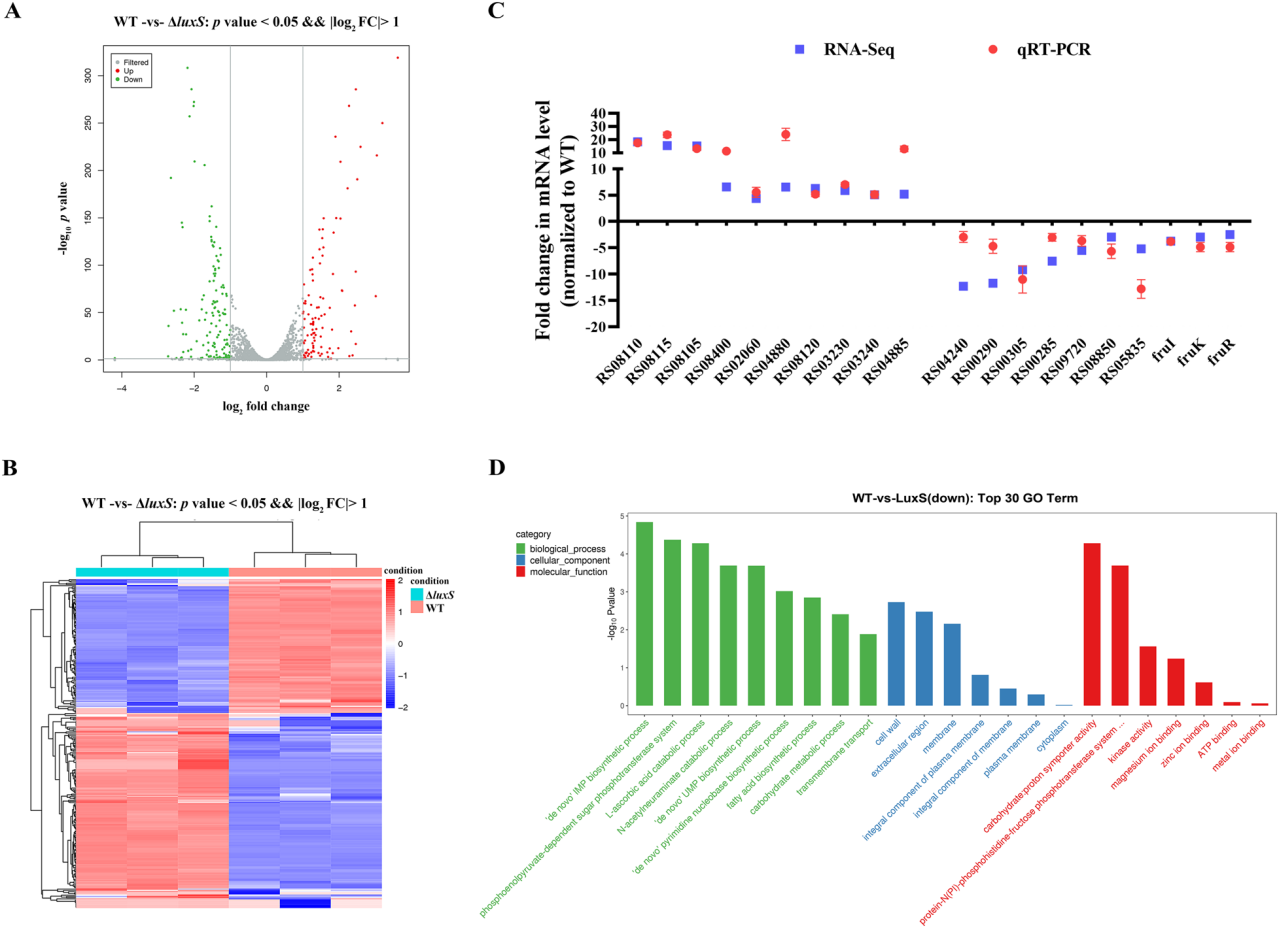
**Comparative transcriptomics analysis.** **A** Volcano plot of the differentially expressed genes. The *x*-axis represents fold change (log_2_) and* y*-axis represents *p*-value (log_10_). Red dots represent upregulated genes and green dots indicate downregulated genes with a significant difference. **B** Heat map of gene expression. A total of 264 genes were differentially expressed in the Δ*luxS* strain compared to the wild-type (WT) strain. Red and blue fonts represent up- and down-regulated genes, respectively in the WT or Δ*luxS* strains. **C** Relative mRNA levels of 20 differently expressed genes determined by RT-qPCR. Data are expressed as n-fold change normalized to mRNA level of WT. **D** Gene Ontology (GO) classification of downregulated genes in the Δ*luxS* strain.

To determine the role of *luxS* in intracellular survival of *S. agalactiae*, we performed RNA transcriptome sequencing (RNA-Seq) for WT and Δ*luxS* mutant strains. Genes with over twofold change ((log_2_ ≤  −1.0 or log_2_ ≥ 1.0) were considered differentially expressed. A total of 264 genes were identified in the Δ*luxS* strain, including 155 upregulated (red) and 109 downregulated (green) genes (Fig. 1A; Additional file 4). The hierarchical clustering clearly illustrated the magnitude difference of the differentially expressed genes between WT and Δ*luxS* (Fig. 1B). To validate the reliability of our RNA-Seq results, RT-qPCR was performed to measure the expression levels of the DFGs. The expression patterns of 20 genes randomly selected (10 up-regulated and 10 down-regulated genes) were consistent with the transcriptomic data (Fig. 1C). Further, we classified the DFGs according to the Gene Ontology (GO) descriptions. The DEGs were primarily classified into biological process, cellular component, and molecular function (Fig. 1D). Specifically, the TOP 30 enriched GO terms (down-regulated in Δ*luxS*) included carbohydrate metabolic process, membrane component and ion transport activity. Notably, the putative fructose metabolic operon (*fruRKI*) was significantly down-regulated in Δ*luxS*, which has been reported to be involved in the intracellular survival of *S. pyogenes* [10]. The enrichment scores, *p*-value and the down-regulated genes contained in the GO terms are listed in Additional file 5.

### *luxS* deficiency decreases carbohydrate metabolism of* S. agalactiae*

**Fig. 2:**
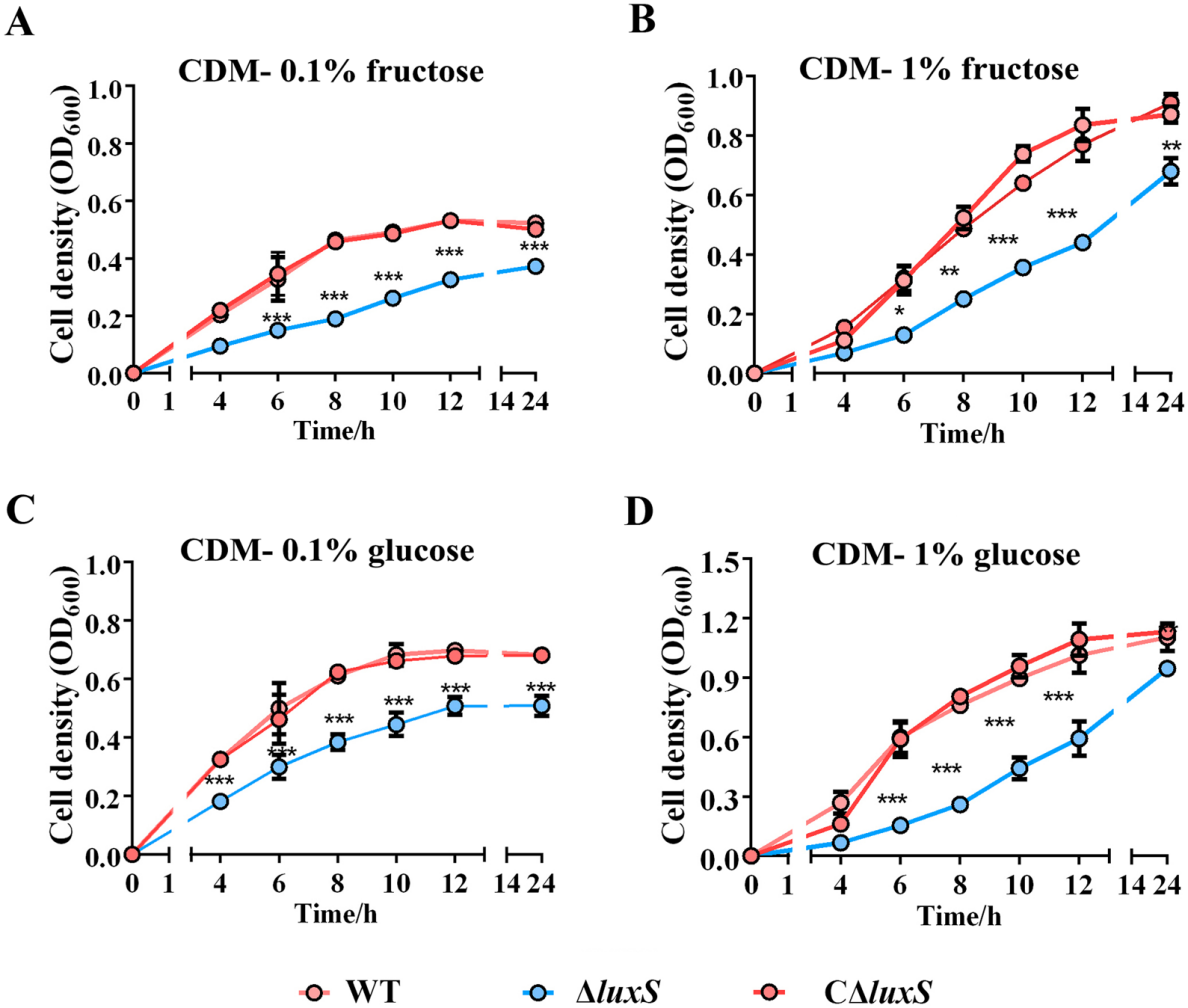
**Influence of luxS deficiency on carbon source availability of S. agalactiae strains.** All strains were cultivated in a chemically-defined medium (CDM) supplemented fructose (**A**, **B**) or glucose (**C**, **D**) at a range of concentrations from 0.1% to 1% (w/v) as the sole carbon source, separately. Data are presented as the mean ± SD for three independent experiments. *P < 0.05, **P < 0.01, or ***P < 0.001, indicates a significant difference between the indicated strain and the WT strain.

Considering the repressed expression of carbohydrate metabolic process-related genes in the Δ*luxS* strain, we sought to ascertain whether *luxS* is involved in the utilization of carbon sources in *S. agalactiae*. As shown in Additional file 6, there was no difference in growth kinetics between the WT and Δ*luxS* strains in THB. When we used CDM supplemented with glucose or fructose as the sole carbon source, the Δ*luxS* strain showed significantly decelerated growth. In CDM supplemented with 0.1% (w/v) (Fig. 2A) or 1% (w/v) (Fig. 2B) fructose, Δ*luxS* showed a significantly lower bacterial cell density as indicated by OD_600_ when compared to the WT or CΔ*luxS* strains at all sampling time points. A similar alteration was observed with supplementation of 0.1% (Fig. 2C) or 1% (Fig. 2D) of glucose.

### *luxS* contributes to intracellular survival of *S. agalactiae* via upregulating the transcription of* fruRKI* operon

**Fig. 3:**
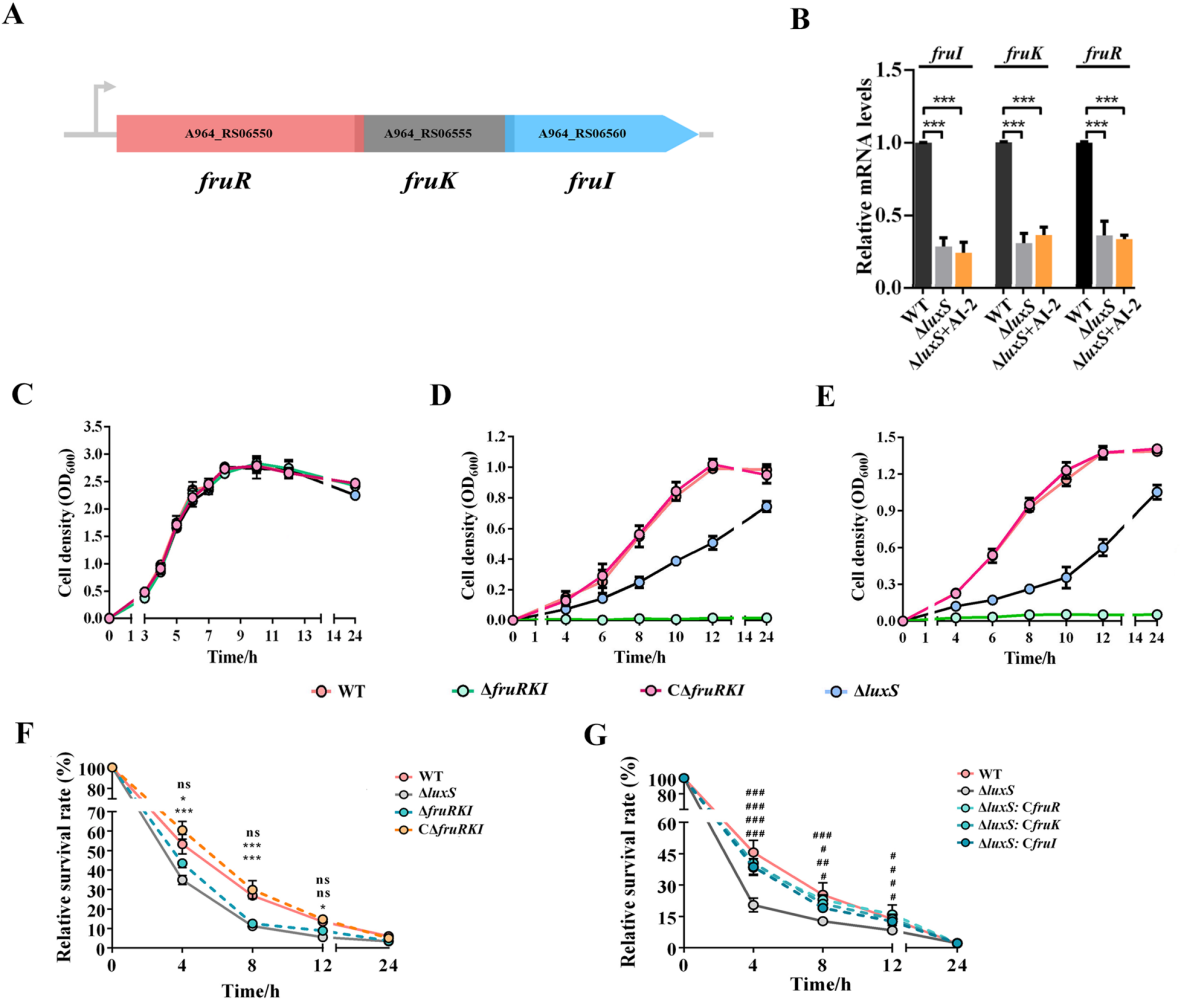
**Growth characters and intramacrophage survival capabilities of S. agalactiae strains.** **A** Genetic structure of the *fruRKI* operon in *S. agalactiae*. **B** Relative mRNA levels of the *fruRKI* genes. **C** Growth curves of the WT, Δ*luxS*, Δ*fruRKI,* and CΔ*fruRKI* strains cultivated in THB. **D** Growth curves of the WT, Δ*luxS*, Δ*fruRKI* and CΔ*fruRKI* strains cultivated in CDM supplemented 1% fructose. **E** Growth curves of the WT, Δ*luxS*, Δ*fruRKI* and CΔ*fruRKI* strains cultivated in CDM supplemented 1% glucose. **F** Intracellular survival of the WT, Δ*luxS*, Δ*fruRKI* and CΔ*fruRKI* strains in macrophages. **G** Intracellular survival of the WT, Δ*luxS*, Δ*luxS*:: *fruI*, Δ*luxS*:: *fruK* and Δ*luxS*:: *fruR* strains in macrophages. Data are presented as the mean ± SD of three independent experiments. *P < 0.05 or ***P < 0.001, indicates a significant difference between the indicated strain and the WT strain. ^#^ < 0.05, ^##^ < 0.01, or ^###^ < 0.001, indicates a significant difference between the indicated strain and the Δ*luxS* strain.

Based on the transcriptomic data, we identified the putative fructose operon *fruRKI* was downregulated in Δ*luxS* mutant strain. In the genome of *S. agalactiae* GD201008-001, *fruR* (encoding the DeoR/GlpR transcriptional regulator), *fruK* (encoding 1-phosphofructokinase) and *fruI* (encoding PTS fructose-specific EIIC) are three contiguous genes with a 3-bp overlap, indicating that they might represent an operon (Fig. 3A). To further support the notion, we isolated total RNA from WT and reverse-transcribed into cDNA as a template and successfully amplified a 3.1 kb transcript between *fruR* and *fruI* (Additional file 7A), indicating that *fruI*, *fruK* and *fruR* genes comprised an operon in *S. agalactiae*. Further, we want to investigate whether AI-2 molecules can negate the significantly down-regulated expression of *fruRKI* caused by *luxS* deficiency*.* As a result, the addition of AI-2 could not effectively improve the mRNA levels of *fruRKI* in the Δ*luxS* strain (Fig. 3B)*.*

To determine whether decreased bacterial intracellular survival caused by *luxS* deficiency was due to the down-regulation of *fruRKI* operon, we constructed the whole *fruRKI* operon knock-out mutant strain Δ*fruRKI* and its complementary strain CΔ*fruRKI*. Before proceeding intracellular assay, we compared the growth characteristics of the WT and Δ*fruRKI* mutant strains in THB or CDM supplemented with 1% fructose (fructose-CDM) or 1% glucose (glucose-CDM). As shown in Fig. 3C, the growth kinetics of Δ*fruRKI* in the THB medium was similar to that of the WT. However, Δ*fruRKI* failed to grow in fructose (Fig. 3D) or glucose (Fig. 3E) as the sole carbon source. The intracellular survival assay was performed by infecting RAW264.7 macrophage cell line, which has been validated as an in vitro platform to evaluate piscine streptococcus-macrophage interactions [12, 13, 24]. As shown in Fig. 3F, at each time point, the number of intracellular viable bacteria in both Δ*luxS* and Δ*fruRKI* mutant strains was significantly decreased compared to that of the WT and CΔ*fruRKI* strains. To further confirm whether the down-regulation of the *fruRKI* operon could be accountable for the decreased intracellular survival of Δ*luxS* mutant strain, we have tried to overexpress the whole *fruRKI* operon in the Δ*luxS* strain, however, the operon is too large to be cloned into the pSET2 vector. Thus, we overexpressed the individual genes in the *fruRKI* operon in the Δ*luxS* strain, and obtained the strains Δ*luxS*:: *fruI*, Δ*luxS*:: *fruK* and Δ*luxS*:: *fruR*. The measurement for the number of surviving bacteria showed that overexpression of any gene located in the *fruRKI* operon significantly restored the viability of Δ*luxS* mutant strain in RAW264.7, although the single restoration could not make the number of viable bacteria reach the level of the WT strain (Fig. 3G).

### *luxS* indirectly regulates the promoter activity of the *fruRKI* operon

**Fig. 4:**
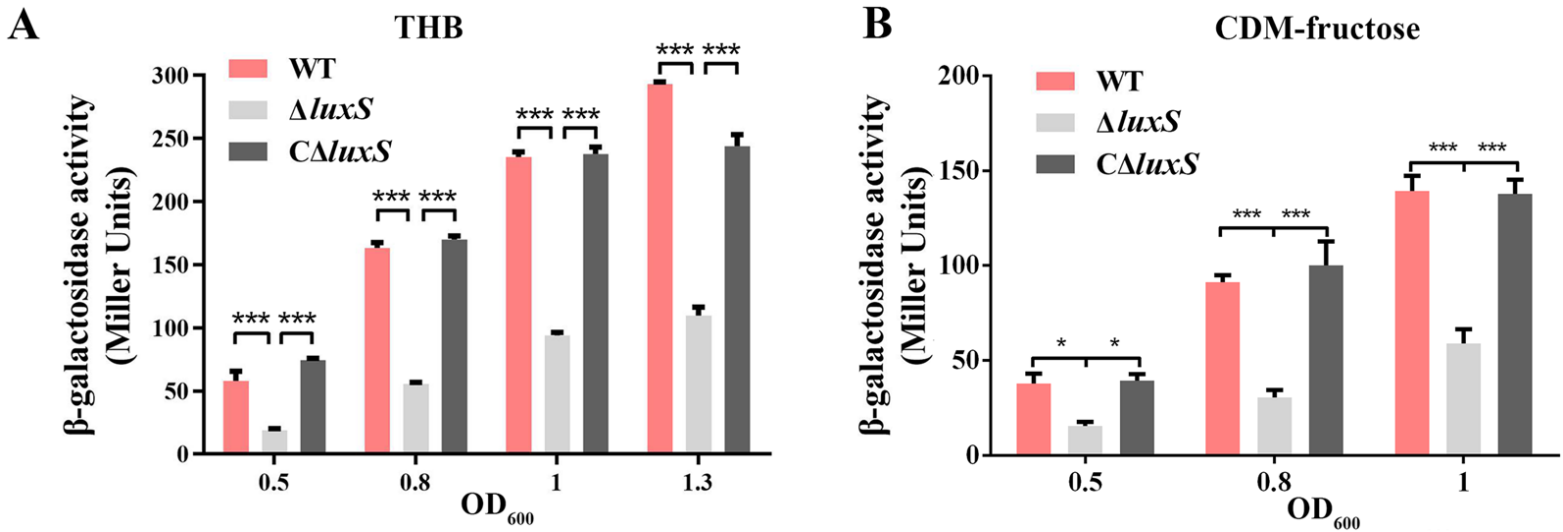
**The promoter activity of fruRKI in S. agalactiae strains grown in rich medium or chemically defined medium (CDM).** **A** The *fruRKI* promoter activity in the WT, Δ*luxS* and CΔ*luxS* strains cultivated in THB (rich medium). **B** The *fruRKI* promoter activity in the WT, Δ*luxS* and CΔ*luxS* strains cultivated in CDM supplemented 1% fructose. Data are presented as the mean ± SD of three independent experiments. ***P < 0.001.

Considering that inactivation of *luxS* downregulated the expression of all three genes located in the *fruRKI* operon as evidenced by the transcriptomic data, we make a conjecture that *luxS* may have affected the transcription of *fruRKI* via modulating the promoter. To determine the transcription level of *fruRKI*, we examined the promoter activity by utilizing a transcriptional fusion of *lacZ* reporter gene with the promoter of *fruRKI* in the WT and Δ*luxS* strains grown in THB or fructose-CDM. At any of the stages of bacterial growth in THB, the *fruRKI* promoter showed decreased activity in the Δ*luxS* strain compared with WT, while the introduction of *luxS* in the Δ*luxS* strain almost completely restored the activity of *fruRKI* promoter (Fig. 4A). A similar result was also observed in the WT and Δ*luxS* strains grown in fructose-CDM (Fig. 4B). Further, we performed the EMSA and confirmed that the LuxS protein could not bind to the promoter of *fruRKI* operon (Additional file 7B).

### A catabolite responsive element (*cre*) is shared by the promoters of* luxS* and *fruRKI* operon

**Fig. 5:**
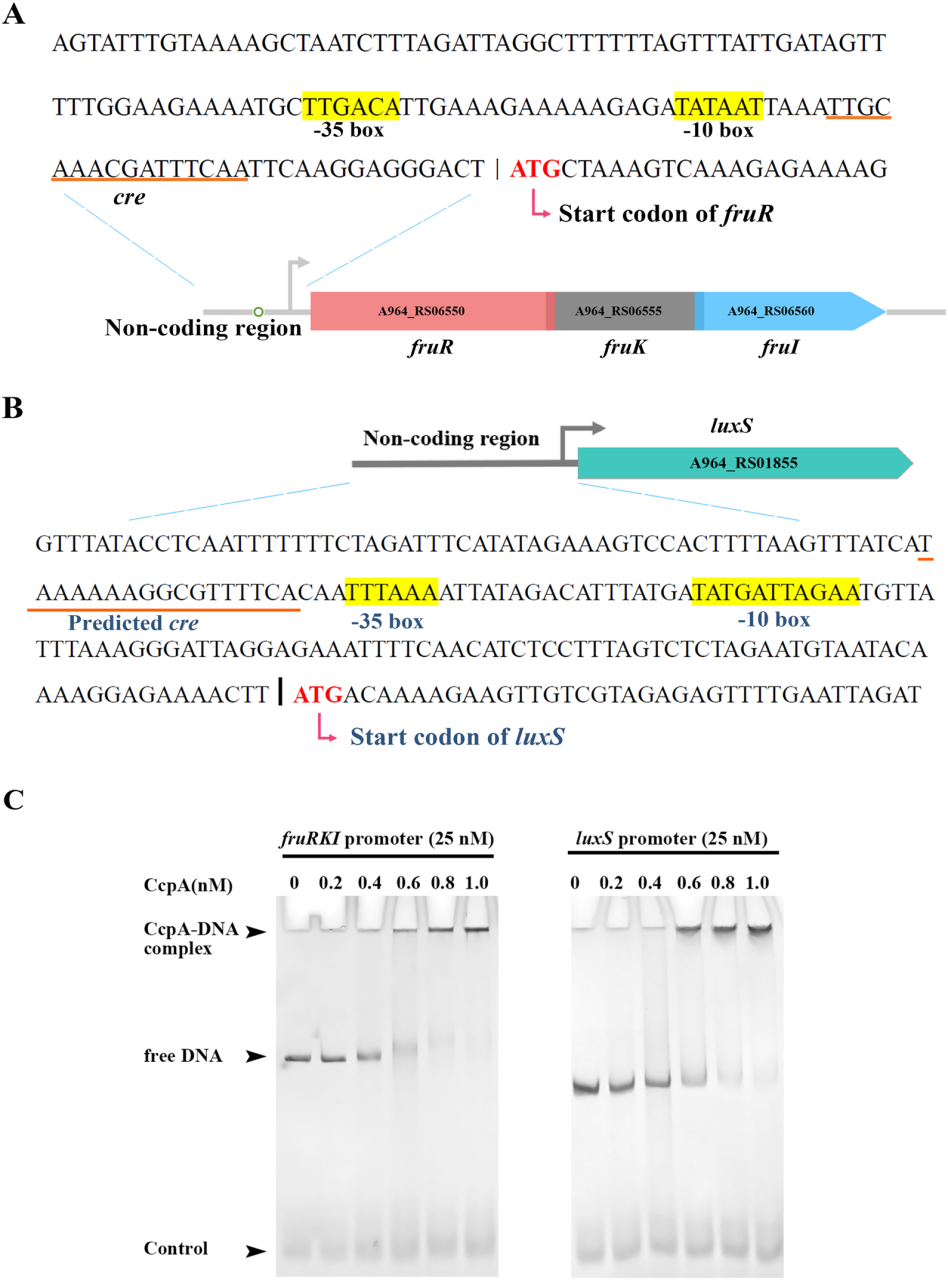
**The in vitro binding of CcpA to the fruRKI promoter or luxS promoter.** **A** Diagrams depicting the noncoding region of the *fruRKI* operon. The magnified region indicates the promoter sequences, including the putative *cre* for CcpA binding (underlined), and the −10 and −35 positions are highlighted and labeled. **B** Diagrams depicting the noncoding region of the *luxS* gene. The putative *cre* is underlined, and the −10 and −35 positions are highlighted and labeled. **C** An electrophoretic mobility shift assay (EMSA) showing the binding ability of CcpA protein to the *fruRKI* or *luxS* promoters (25 nM). The concentrations of CcpA ranged from 0 to 1.0 μM.

The CcpA has previously been reported to be the major regulator involved in controlling carbon-metabolism by binding to the cis-regulatory element *cre* to activate or repress the transcription of target genes in Gram-positive bacteria [28]. In this study, a cis-acting sequence was identified in the *fruRKI* promoter (Fig. 5A). Interestingly, a putative *cre* was also found in the promoter region of *luxS* (Fig. 5B). This shared feature by the two promoters led us to hypothesize that CcpA may play a certain role in the relationship between *luxS* and *fruRKI*. To illustrate this point, we first performed the EMSA using purified CcpA protein with the DNA fragments containing the promoter of *fruRKI* or *luxS*. As shown in Fig. 5C, CcpA could directly bind to the promoter regions of both *fruRKI* and *luxS*. This result indicated that both *luxS* and *fruRKI* transcription were directly regulated by CcpA. Further, we have tried to delete *ccpA* from *S. agalactiae* using homologous recombination, but unfortunately, all our attempts failed, suggesting that this gene might indeed be essential for bacterial survival. Then we performed RT-qPCR to detect the transcription level of *ccpA* in the WT and Δ*luxS* strains. No significant difference was observed in the *ccpA* transcription between the two bacterial strains (Additional file 8). In addition, our transcriptomic data also confirmed that the deletion of *luxS* does not result in altered *ccpA* expression. These findings exclude the possibility that loss of *luxS* results in the upregulation of CcpA, thereby repressing the expression of the *fruRKI* operon.

### Deletion of *luxS *promotes the binding of CcpA to the *fruRKI* promoter

**Fig. 6:**
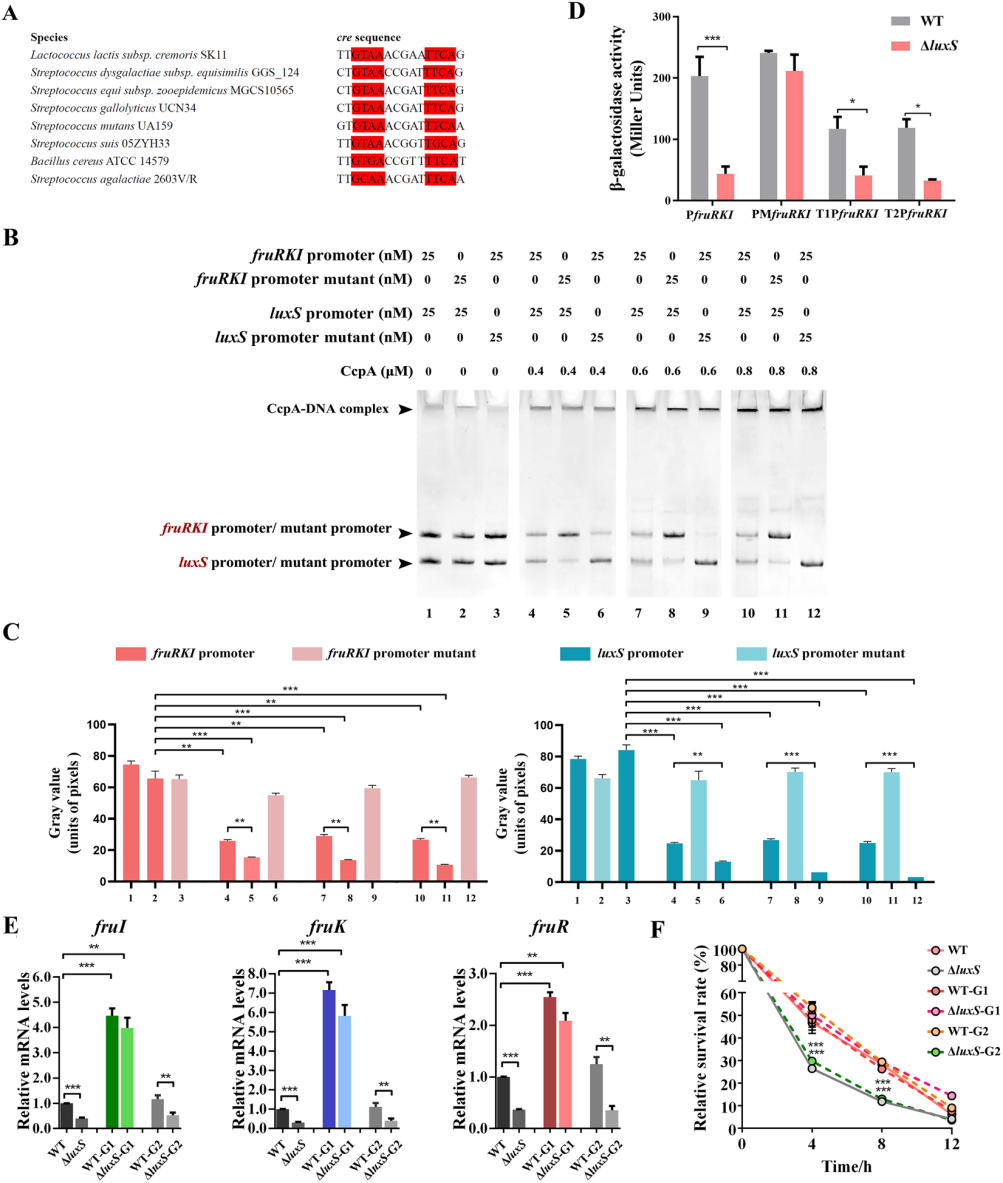
***luxS* indirectly regulates the expression of* fruRKI* operon through the* cre* region in the* fruRKI* promoter**. **A** The sequence alignment of *cre* from eight gram-positive bacteria. The conserved regions of *cre* are highlighted. **B** Competitive binding of the *luxS* promoter and the *fruRKI* promoter to CcpA by EMSA analysis. The 129-bp fragment of *luxS* promoter was tested for its ability to bind with CcpA (0.4, 0.6 and 0.8 μM) in presence of 188-bp fragment of wild type *fruRKI* promoter (lane 4, 7 and 10) or *fruRKI* promoter with mutations on *cre* (lane 6, 9 and 12); the 188-bp fragment of *fruRKI* promoter was tested for its ability to bind with CcpA (0.4, 0.6 and 0.8 μM) in presence of 129-bp fragments of wild type *luxS* promoter (lane 4, 7 and 10) or *luxS* promoter with mutations on *cre* (lane 5, 8 and 11); Lane 1 to 3. Different components of internal controls. **C** The DNA-binding capacity of CcpA was measured with grayscale analysis of the blots. **D** The *fruRKI* promoter activity in the WT and Δ*luxS* strains containing the PM*fruRKI*-lacZ reporter plasmid. The WT and Δ*luxS* strains containing T1P*fruRKI*-lacZ or T2P*fruRKI*-lacZ reporter plasmids serve as control groups. The *β*-galactosidase activity was expressed as relative miller units. **E** Relative mRNA levels of the *fruRKI* genes determined by real-time PCR. WT-G1: eight-base substitution in the *cre* conserve region of *fruRKI* promoter in WT; Δ*luxS*-G1: eight-base substitution in the *cre* conserve region of *fruRKI* promoter in Δ*luxS*; WT-G2: eight-base substitution in the *cre* non-conserve region of *fruRKI* promoter in WT; Δ*luxS*-G2: eight-base substitution in the *cre* non-conserve region of *fruRKI* promoter in Δ*luxS*. **F** Intracellular survival rates of the WT, Δ*luxS*, WT-G1, Δ*luxS*-G1, WT-G2 and Δ*luxS*-G2 strains in macrophages. Data are presented as the mean ± SD of three independent experiments. *P < 0.05, **P < 0.01, or ***P < 0.001.

To determine whether the reduced transcription level of the *fruRKI* gene in Δ*luxS* is due to the increased binding ability between CcpA and the *fruRKI* promoter, we mutated the conserved nucleotides of *cre* in the *luxS* and *fruRKI* promoter regions, respectively, and tested their binding capacity to CcpA protein by EMSA. According to the RegPrecise Database, the *cre* regions from eight species of Gram-positive bacteria were aligned and a total of eight nucleotides were identified to be most conserved (Fig. 6A). To confirm mutation of these conserved nucleotides on *cre* abolishes the binding of CcpA, we amplified the *luxS* and *fruRKI* promoter regions containing the *cre* and *cre* mutant (denoted as *luxS* mutant promoter and *fruRKI* mutant promoter), respectively. The EMSA was performed with four groups of different concentrations of CcpA protein (Fig. 6B). The EMSA showed that the mutation of *cre* in either *luxS* promoter or *fruRKI* promoter reduced detectable binding of respective promoters to CcpA (Fig. 6B and C). Results of β-galactosidase activity assay revealed that the P*fruRKI* activity was remarkably decreased in Δ*luxS* compared to the WT strain (Fig. 6D), but no significant difference was observed in promoter activity between Δ*luxS* and WT after mutating the *cis*-acting sequences of *fruRKI* promoter (PM*fruRKI*). Moreover, the activities of T1P*fruRKI* and T2P*fruRKI* were not affected due to mutation in non-*cis* region of the *fruRKI* promoter. To determine the importance of *cre* in the regulation of *fruRKI* transcription by *luxS*, we mutated the *cre* region of *fruRKI* promoter in the WT and Δ*luxS* strains. The resulting mutant strains were named WT-G1 and Δ*luxS*-G1, respectively. As expected, transcription of *fruRKI* in WT-G1 was significantly enhanced compared with the WT strain while no significant difference was seen between the WT-G1 and Δ*luxS*-G1 strains (Fig. 6E). Overall, our results indicate that deletion of *luxS* leads to increased binding of CcpA to the *fruRKI* promoter.

In addition, to determine whether reduced intracellular survival ability exhibited by the Δ*luxS* strain can be attributed to *cre-*mediated downregulation of the *fruRKI* operon, we infected the RAW264.7 macrophages with WT, Δ*luxS*, WT-G1 and Δ*luxS*-G1 strains. The WT-G1 incubated with RAW264.7 showed a comparable ability in intracellular survival to that of the WT, whereas the Δ*luxS* mutant exhibited a significant decrease during the entire experiment (Fig. 6F). Importantly, the survival ability of Δ*luxS*-G1 was completely restored to the WT level (Fig. 6F).

## Discussion

Our understanding of *luxS* has been predominantly focused on its role in quorum sensing. Here, we demonstrated that *luxS* also affected the ability of *S. agalactiae* to survive within macrophages by regulating the transcription of the carbohydrate utilization operon *fruRKI*. Using site-directed mutagenesis, we demonstrated that the *cre* located in the promoter of *fruRKI* operon is a critical locus for *luxS* to play an indirect regulatory role. Our study reveals a new mechanism by which *S. agalactiae* can adapt their metabolism to the available nutrients in macrophages and thus survive efficiently.

*S. agalactiae* could withstand the extreme environment in macrophages and persist inside the fully mature phagolysosome for a relatively long period [8]. We have previously found that *luxS* deficiency decreased the intracellular survival ability of *S. agalactiae* but this weakening effect was not mediated by the signaling molecule AI-2 [13]. LuxS has been reported to be involved in many cellular processes, but little is known about its function in intracellular survival. LuxS is required for AI-2 biosynthesis, but it also plays a crucial role in the activated methyl cycle (AMC), which is involved in the utilization of S-adenosylmethionine (SAM) [29–31]. In *Streptococcus sanguinis*, *luxS* deletion has resulted in a large number of gene expression changes due to the accumulation of intermediates of SAM metabolism [32]. Therefore, we speculate that reduced intracellular survival ability in the Δ*luxS* might be linked to the altered gene expression pattern. To verify this, we performed a comparative transcriptomics analysis of the WT and Δ*luxS* strains and identified 264 differentially expressed genes. Interestingly, the genes involving carbohydrate metabolisms, such as sugar-specific phosphotransferase system (PTS) and carbohydrate ABC transporter, account for a large proportion of downregulated transcripts.

One previous study has revealed that *S. agalactiae* genomes lack genes involved in the biosynthesis of the tricarboxylic acid cycle (TCA) [33]. However interestingly, Patron et al. [34] reported that this bacterium has a broad ability to import carbohydrate sources to adapt to the host environment. Therefore, we speculated that the utilization of carbohydrates may be essential for *S. agalactiae* survival within phagocytes. In this study, the downregulation of the *fruRKI* operon in the *luxS* mutant of *S. agalactiae* attracted our attention. The *fruRKI* operon has been demonstrated in *S. mutans* to play important roles in various biological processes including sugar metabolism and biofilm formation [35]. Additionally, the operon *fruRBA,* similar to *fruRKI*, has been shown to be required for *S. pyogenes* growth in fructose and for resistance to neutrophil killing in human blood [10]. Our present study showed that the growth of Δ*luxS* exhibited significant weakening in a chemically defined medium with a low concentration of fructose or glucose, and the transcription of *fruRKI* was also significantly lower than that of the WT strain. Furthermore, we also demonstrated that the *fruRKI* operon was important for *S. agalactiae* intracellular survival. Accordingly, it is safe to reach the conclusion that downregulation of the *fruRKI* operon in the Δ*luxS* strain results in deficiencies in carbohydrate metabolism, thereby reducing the survival of this bacterium within nutrient-poor macrophages [36]. Notably, downregulation in the mRNA level of *fruRKI* could not be eliminated by the addition of exogenous AI-2, which further gives support to our previous finding that LuxS contributes to intracellular survival of *S. agalactiae* independent of the effect of AI-2 [13].

It is widely known that pathogens utilize carbon catabolite repression (CCR) to effectively assimilate a preferred carbon in response to local differences in nutrient availability within the host [28]. CcpA is the master transcriptional regulator of CCR, which can repress or activate gene transcription by binding *cis*-acting DNA known as the *cre* [37, 38]. In this study, we identified a putative *cre* in the promoters of *fruRKI*, which has been demonstrated to be directly bound by CcpA, thereby inhibiting *fruRKI* transcription in *Lactococcus lactis* [39]. Consequently, an intriguing question arose: can CcpA transcription be regulated by LuxS? If loss of *luxS* results in an increase in CcpA transcript levels, then it can explain our observations of repression of *fruRKI* operon. However, our study provides evidence that *ccpA* transcription has not been altered due to the inactivation of *luxS*, and therefore is not responsible for the downregulated expression of *fruRKI* in the Δ*luxS* strain.

We have attempted to verify the bridge role of CcpA between *luxS* and *fruRKI* by deleting the *ccpA* gene from *S. agalactiae*, but regretfully, we failed to obtain the *ccpA* deletion mutant. This gene has been reported to be essential for *S. agalactiae* survival [40]. However, Roux et al. [41] deleted the *ccpA* gene in *S. agalactiae* strain A909 successfully. No more than 95% query cover exist between the genomes of the piscine GD201008-001 and human-derived isolate A909, which might explain the contradiction between different researches. Interestingly, knockout of *luxS* caused a significantly reduced activity of the *fruRKI* promoter. And the *cre* in the promoter of *fruRKI* operon was determined to be an action target region of *luxS* since its nucleotide substitutions eliminated the effect of *luxS* on *fruRKI* operon and restored the intracellular survival ability of the Δ*luxS* strain. All the above findings suggest that the altered binding capability of CcpA to the *cre* in the Δ*luxS* strain accounts for decreased *fruRKI* expression. Based on the evidence presented here, we propose a model for the regulation between *luxS*-*fruRKI*-CcpA (Additional file 9). In short, the deletion of *luxS* enhances the binding of CcpA to the *cre* of the *fruRKI* promoter, resulting in reduced *fruRKI* transcription. Whilst our study has highlighted the important role that the recognition of *cre* by CcpA plays in affecting the transcription of *fruRKI* operon by *luxS*, it remains unclear how *luxS* deficiency exerts an effect on the binding capability of CcpA to the *cre* in the *luxS* promoter. In addition, we also cannot rule out the possibility that proteins other than CcpA that have the ability to bind this *cre* responsive element may contribute to the changes seen in this study, although only CcpA has so far been reported to preferentially bind to the *cre*. Future work will clarify these issues.

Notably, according to the RegPrecise database, the *cre* regions were found in the promoters of 23 genes related to carbohydrate metabolism (Additional file 10), representing 74.19% of the downregulated genes. To determine whether our findings relating to CcpA are specific to the *fruRKI* promoter, we randomly selected another two promoters (P*ptsG* and P*rbsR*) containing the *cre* to perform competitive EMSA. Unsurprisingly, they could also bind to CcpA competitively with *luxS* (Additional file 11). This finding suggests that the regulatory role of *luxS* in carbohydrate metabolism may be broad by affecting the binding of *cre* to CcpA. But it is important to realize that not all carbohydrate metabolism-related genes with the *cre* element could be regulated by *luxS*. For example, a conserved PTS *manLMN* operon was not transcriptionally affected by *luxS* deficiency based on our RNA-seq data and subsequent RT-qPCR validation (data not shown). We speculate that there may be unknown regulators involved in *manLMN* transcription. In *S. mutans*, the *manLMN* operon is regulated not only by CcpA but also by FruR and EIIMan [34].

In conclusion, our findings establish, for the first time*,* a link between *luxS* and intracellular survival of *S. agalactiae*, which advances our understanding of the *luxS* function in mediating bacterial pathogenesis. Further studies providing insight into the precise mechanism of *luxS* effect may uncover novel therapeutic avenues.

### Supplementary Information

**Additional file 1. Bacterial strains and plasmids.**

**Additional file 2. Primers used in this study.**

**Additional file 3. Primer used for qRT-PCR.**

**Additional file 4. The differentially expressed genes in Δ*luxS*** **compared with wild-type strain.**

**Additional file 5. The GO enrichment analysis of down-regulated gene in Δ*luxS*** **compared with wild-type strain.**

**Additional file 6. The growth of the WT, Δ*luxS*** **and CΔ*luxS*** **strains in THB.**

**Additional file 7. LuxS protein could not bind to the promoter of *fruRKI* operon. **(A) The fruRKI operon was identified in the genome of S. agalactiae GD201008-001. Lane 1. A fragment amplified by PCR from the cDNA obtained by reverse trancription. Lane 2. A fragment amplified by PCR from genomic DNA as the positive control. M. DNA marker. (B) Binding ability of LuxS protein to the fruRKI promoter. Lane 1. Negative control (25 nM of fruRKI promoter). Lane 2–4. Positive controls. Binding reaction to 25 nM of fruRKI promoter with CcpA protein at a range of concentrations from 0.6 to 1 μM. Lane 5–7. Binding reaction to 50 nM of fruRKI promoter with LuxS protein at a range of concentrations from 1 to 3 μM.

**Additional file 8. Relative mRNA levels of *ccpA* gene by real-time PCR.**

**Additional file 9. The model for the the regulation between *luxS***-***fruRKI*-CcpA.**

**Additional file 10. The *cre*** **regions in the promoters of the downregulated genes in RNA-seq.**

**Additional file 11. Competitive EMSA analyses the binding of CcpA to** ***ptsG* (A) or *rbsR* (B) promoters.**

## Data Availability

The RNA-Seq data generated from this study were submitted to the NCBI Sequence Read Archive (SRA) under accession numbers SRR16885436 to SRR16885441.
