# Supplementary material for: luxS contributes to intramacrophage survival of Streptococcus agalactiae by positively affecting the expression of fruRKI operon
Source: Vet Res. 2023 Sep 27;54:83. doi: 10.1186/s13567-023-01210-9 (PMC10536698; doi:10.1186/s13567-023-01210-9)
Supplement: Supplementary file 1 — Additional file 1. Bacterial strains and plasmids. [file 13567_2023_1210_MOESM1_ESM.docx]

**Additional file 1. Bacterial strains and plasmids**

| **Strain or plasmid** | **Description** | **Source or reference** |
| --- | --- | --- |
| Strains |  |  |
| *S. agalactiae* GD201008-001 serotype Ia strain | A virulent strain of *S. agalactiae* isolated from tilapia with meningoencephalitis in China | [21] |
| Δ*luxS* mutant | *luxS* gene deletion mutant of GD201008-001 | [13] |
| Δ*fruRKI* mutant | *fruRKI* operon deletion mutant of GD201008-001 | This study |
| Δ*luxS* complemented mutant (CΔ*luxS*) | GD201008-001 Δ*luxS* mutant complemented with vector pSET2-*luxS*; Spc^r^ | [13] |
| Δ*luxS* complemented *fruI* (Δ*luxS*::*fruI*) | GD201008-001 Δ*luxS* mutant complemented with vector pSET2-*fruI*; Spc^r^ | This study |
| Δ*luxS* complemented *fruK* (Δ*luxS*::*fruK*) | GD201008-001 Δ*luxS* mutant complemented with vector pSET2-*fruK*; Spc^r^ | This study |
| Δ*luxS* complemented *fruR* (Δ*luxS*::*fruR*) | GD201008-001 Δ*luxS* mutant complemented with vector pSET2-*fruR*; Spc^r^ | This study |
| *S. agalactiae* GD201008-001 cre on fruRKI promoter allelic replacement strain (WT-G1) | Eight nucleotides in cre of *fruRKI* promoter replaced mutant of GD201008-001. | This study |
| Δ*luxS* mutant *cre* on *fruRKI* promoter allelic replacement strain (Δ*luxS*-G1) | Eight nucleotides in cre of *fruRKI* promoter replaced mutant of Δ*luxS*. | This study |
| *S. agalactiae* GD201008-001 *fruRKI* promoter allelic replacement strain (WT -G2) | Eight nucleotides in *fruRKI* promoter replaced mutant of GD201008-001. | This study |
| *S. agalactiae* GD201008-001 *fruRKI* promoter allelic replacement strain (*luxS* -G2) | Eight nucleotides in fruRKI promoter replaced mutant of Δ*luxS*. | This study |
| *E. coli* DH5α | Used for production of recombinant plasmids | Invitrogen |
| *E. coli* BL21 | Used for protein expression | Invitrogen |
| Plasmids |  |  |
| pSET4S | Thermosensitive suicide vector for gene replacement in *Streptococcus* replication of pG+ host3 and pUC19; lacZ’ Spc^r^ | [23] |
| pSET2 | *E. coli-Streptococcus* shuttle vector; Spc^r^ | [25] |
| pTCV-lac | Shuttle vector carrying promoterless *lacZ* gene for *β*-galactosidase assay; Km^r^ | [26] |
| P*fruRKI*-lacZ | Recombinant vector with pTCV-lac background, designed for *fruRKI* promoter activities assay; Km^r^ | This study |
| PM*fruRKI*-lacZ | Recombinant vector with pTCV-lac background, designed for promoter activities assay of 8 points mutations at cre on fruRKI promoter; Km^r^ | This study |
| T1P*fruRKI*-lacZ | Recombinant vector with pTCV-lac background, designed for promoter activities assay of 8 points mutations at sites other than cre on fruRKI promoter; Km^r^ | This study |
| T2P*fruRKI*-lacZ | Recombinant vector with pTCV-lac background, designed for promoter activities assay of 8 points mutations at sites other than cre and T1 on fruRKI promoter; Km^r^ | This study |
| pSET2-*fruR* | Complementation vector with pSET2 background containing the promoter followed by full-length *fruR* ORF; Spc^r^ | This study |
| pSET2-*fruK* | Complementation vector with pSET2 background containing the promoter followed by full-length *fruK* ORF; Spc^r^ | This study |
| pSET2-*fruI* | Complementation vector with pSET2 background containing the promoter followed by full-length *fruI* ORF; Spc^r^ | This study |
| pET 32a | Usd for cloning and high-level expression of peptide sequences fused with the 109aa Trx•Tag™ thioredoxin protein; Amp^r^ | Novagen |
| pET 32a-*ccpA* | Recombinant vector with pET 32a background, designed for expresses CcpA; Amp^r^ | This study |
