# Supplementary material for: luxS contributes to intramacrophage survival of Streptococcus agalactiae by positively affecting the expression of fruRKI operon
Source: Vet Res. 2023 Sep 27;54:83. doi: 10.1186/s13567-023-01210-9 (PMC10536698; doi:10.1186/s13567-023-01210-9)
Supplement: Supplementary file 2 — Additional file 2. Primers used in this study. [file 13567_2023_1210_MOESM2_ESM.docx]

**Additional file 2. Primers used in this study**

| **Primer** | **Sequence (5′–3′)** | **Restriction site** | **Function** |
| --- | --- | --- | --- |
| *fruRKI*-1 | GAGCTCGGTACCCGGGGATCCAAAATGATAAACCCAACGAAA | BamH I | Left arm of *fruRKI*; amplifies the flank sequence located in coding region of *fruRKI* upstream (556 bp) |
| *fruRKI*-2 | AGGAGGGACTTTAATCTCAACTTATCCAAATTTAC |  |  |
| *fruRKI*-3 | TTGAGATTAAAGTCCCTCCTTGAATTGAAA |  | Right arm of *fruRKI*; amplifies the flank sequence located in coding region of *fruRKI* upstream (506 bp) |
| *fruRKI*-4 | CAGGTCGACTCTAGAGGATCCTGAAACACGACGACAAGAAG | BamH I |  |
| *fruRKI*-F | GATTGCACGAGCTGGGTC |  | fragment for *fruRKI* ORF; used to amplifies the *fruRKI* operon (3100 bp). |
| *fruRKI*-R | TTGATGCCGGAACTACGAC |  |  |
| *fruI*-1 | GAGCTCGGTACCCGGGGATCCAAAATGATAAACCCAACGAAA | BamH I | Left arm of *fruI*; amplifies the flank sequence located in coding region of *fruI* upstream (556 bp) |
| *fruI*-2 | TAGAAAAATTTTAATCTCAACTTATCCAAATTTAC |  |  |
| *fruI*-3 | TTGAGATTAA AATTTTTCTACCTCAACTTTATTAT |  | Right arm of *fruI*; amplifies the flank sequence located in coding region of *fruI* upstream (456 bp) |
| *fruI*-4 | CAGGTCGACTCTAGAGGATCCTAAAAAGACAGGAGCGGAA | BamH I |  |
| *fruR*-1 | GAGCTCGGTACCCGGGGATCCGAAGGCGTATCGTAAGTTTAG | BamH I | Left arm of *fruR*; amplifies the flank sequence located in coding region of *fruR* upstream (511 bp) |
| *fruR*-2 | AGGAGGGACTATGATTTATACAGTAACATTAAATC |  |  |
| *fruR*-3 | TATAAATCATAGTCCCTCCTTGAATTGA |  | Right arm of *fruR*; amplifies the flank sequence located in coding region of *fruR* upstream (555 bp) |
| *fruR*-4 | CAGGTCGACTCTAGAGGATCCCTAAGTATCATAGGCAACGC | BamH I |  |
| C*fruRKI*-F | GAGCTCGGTACCCGGGGATCCCTATTTTGTTTTACGGAACAAG | BamH I | A fragment for genomic complementation of *fruRKI* operon (3679 bp) |
| C*fruRKI*-R | CAGGTCGACTCTAGAGGATCCATGCTTGACATTGAAAGAAAA | BamH I |  |
| C*fruI*-1 | GAGCTCGGTACCCGGGGATCCAGTATTTGTAAAAGCTAATCTTTAG | BamH I | A fragment of *fruRKI* promoter; amplifies the region of the *fruRKI* promoter (130 bp) |
| C*fruI*-2 | GGATTTTCATAGTCCCTCCTTGAATTGAA |  |  |
| C*fruI*-3 | AGGAGGGACTATGAAAATCCAAGACCTACTAAA |  | A fragment for complementation of *fruI*; amplifies the structural gene of the *fruI* gene (1965 bp) |
| C*fruI*-4 | CAGGTCGACTCTAGAGGATCCCTATTTTGTTTTACGGAACAAG | BamH I |  |
| C*fruK*-1 | GAGCTCGGTACCCGGGGATCCAGTATTTGTAAAAGCTAATCTTTAG | BamH I | A fragment of *fruRKI* promoter; amplifies the region of the *fruRKI* promoter (130 bp) |
| C*fruK*-2 | TATAAATCATAGTCCCTCCTTGAATTGAA |  |  |
| C*fruK*-3 | AGGAGGGACTATGATTTATACAGTAACATTAAATC |  | A fragment for complementation of *fruK*; amplifies the structural gene of the *fruK* gene (912 bp) |
| C*fruK*-4 | CAGGTCGACTCTAGAGGATCCTCATAATTTTTCTACCTCAACTT | BamH I |  |
| C*fruR*-F | GAGCTCGGTACCCGGGGATCCAGTATTTGTAAAAGCTAATCTTTAG | BamH I | A fragment for complementation of *fruR*; amplifies the structural gene of the *fruR* gene, including its own promoter (874 bp) |
| C*fruR*-R | CAGGTCGACTCTAGAGGATCCTCATACTTGAATCACCTTCATT | BamH I |  |
| fruPM-1 | GAGCTCGGTACCCGGGGATCCTGTCAACCGTTATCTGCC | BamH I | Left arm of *fruRKI* promote*r*; amplifies the flank sequence located in coding region of *fruR* upstream (496 bp) |
| fruPM-2 | GAGAAATTAA ATGCTAAAGTCAAAGAGAAAAG |  |  |
| fruPM-3 | ACTTTAGCAT TTAATTTCTCTTCAAAACCTTT |  | Right arm of *fruRKI* promoter; amplifies the flank sequence located in coding region of *fruR* upstream (518 bp) |
| fruPM-4 | CAGGTCGACTCTAGAGGATCCGAGAATACGACCATAACTTGC | BamH I |  |
| TP-G1-1 | GACAGGACGAGCTTTGGT |  | Left arm of points mutations template; amplifies the flank sequence located in *cre* loci of *fruRKI* upstream (824 bp) |
| TP-G1-2 | GTCCATCGTGGTAAATTTAATTATATCTCTTTTTCTTTC |  |  |
| TP-G1-3 | TTTACCACGATGGACATTCAAGGAGGGACTATGC |  | Right arm of points mutations template; amplifies the flank sequence located in *cre* loci of *fruRKI* downstream (634 bp) |
| TP-G1-4 | CACCGATAAAACCTGTAGCA |  |  |
| TP-G2-1 | AATCAAGGTTCGCGTTCT |  | Left arm of points mutations template; amplifies the flank sequence upstream of located in sites other than *cre* of *fruRKI* (758 bp) |
| TP-G2-2 | TCTCTTGGGAGGGCAATGTCAAGCATTTTCTTCCAA |  |  |
| TP-G2-3 | ATTGCCCTCCCAAGAGATATAATTAAATTGCAAACGATTT |  | Right arm of points mutations template; amplifies the flank sequence downstream of located in sites other than *cre* of *fruRKI* (961 bp) |
| TP-G2-4 | CACCGATAAAACCTGTAGCA |  |  |
| P*fruRKI*-lacZ-F | CAAATGAATTCCCGGGGATCCAGTATTTGTAAAAGCTAATCTTTAG |  | A fragment for *fruRKI* promoter fusion on pTCV-lac; amplifies the intergenic spacer region of the *fruRKI* operon (129 bp) |
| P*fruRKI*-lacZ-R | GTATCAACAAGCTGGGGATCCGTCCCTCCTTGAATTGAAA |  |  |
| P*luxS*-lacZ-F | CAAATGAATTCCCGGGGATCCAAGTTTTCTCCTTTTGTATTA |  | A fragment for *luxS* promoter fusion on pTCV-lac; amplifies the intergenic spacer region of the *luxS* gene (188 bp) |
| P*luxS*-lacZ-R | GTATCAACAAGCTGGGGATCCGTTTATACCTCAATTTTTTTCTAG |  |  |
| TP-PM*fruRKI*-1 | GAGAATACGACCATAACTTGC |  | Left arm of points mutations template; amplifies the flank sequence located in *cre* upstream (470 bp) |
| TP-PM*fruRKI*-2 | GTCCATCGTGGTAAATTTAATTATATCTCTTTTTCTTTC |  |  |
| TP-PM*fruRKI*-3 | TTTACCACGATGGACATTCAAGGAGGGACTATGCT |  | Right arm of points mutations template; amplifies the flank sequence located in *cre* downstream (540 bp) |
| TP-PM*fruRKI*-4 | CTTTGTCAACCGTTATCTGC |  |  |
| TP-T1P*fruRKI*-1 | AGTATCATAGGCAACGCTCTG |  | Left arm of points mutations template; amplifies the flank sequence upstream of located in sites other than *cre* (507 bp) |
| TP-T1P*fruRKI*-2 | TCTCTTGGGAGGGCAATGTCAAGCATTTTCTTCCAA |  |  |
| TP-T1P*fruRKI*-3 | ATTGCCCTCCCAAGAGATATAATTAAATTGCAAACGATTT |  | Right arm of points mutations template; amplifies the flank sequence downstream of located in sites other than *cre* (452 bp) |
| TP-T1P*fruRKI*-4 | TCCTCCGATAATGATTGTTTT |  |  |
| TP-T2P*fruRKI*-1 | TGAAACACGACGACAAGAA |  | Left arm of points mutations template; amplifies the flank sequence upstream of located in sites other than *cre* and T1 (490 bp) |
| TP-T2P*fruRKI*-2 | ATGAACTCCTGTCCTTGAAATCGTTTGCAATTTAA |  |  |
| TP-T2P*fruRKI*-3 | AAGGACAGGAGTTCATATGCTAAAGTCAAAGAGAAAA |  | Right arm of points mutations template; amplifies the flank sequence downstream of located in sites other than *cre* and T1 (499 bp) |
| TP-T2P*fruRKI*-4 | CTTTGTCAACCGTTATCTGC |  |  |
| PM*fruRKI*-F | AGTATTTGTAAAAGCTAATCTTTAG |  | *fruRKI* promoter fragment for EMSA; amplifies the intergenic spacer region of the *fruRKI* operon (129 bp) |
| PM*fruRKI*-R | GTCCCTCCTTGAATTGAAA |  |  |
| PM*luxS*-F | AAGTTTTCTCCTTTTGTATTA |  | *luxS* promoter fragment for EMSA; amplifies the intergenic spacer region of the *luxS* gene (188 bp) |
| PM*luxS*-R | GTTTATACCTCAATTTTTTTCTAG |  |  |
| 32aCcpA-F | GCTGATATCGGATCCGAATTCATGAATACAGATGATACGATTAC | EcoRI | A fragment for *ccpA* ORF; used to amplifies the *ccpA* gene (1005 bp) |
| 32aCcpA-R | TTGTCGACGGAGCTCGAATTCCTAATTATTTGTTGTGCCAC | EcoRI |  |
