# Supplementary material for: luxS contributes to intramacrophage survival of Streptococcus agalactiae by positively affecting the expression of fruRKI operon
Source: Vet Res. 2023 Sep 27;54:83. doi: 10.1186/s13567-023-01210-9 (PMC10536698; doi:10.1186/s13567-023-01210-9)
Supplement: Supplementary file 3 — Additional file 3. Primer used for qRT-PCR. [file 13567_2023_1210_MOESM3_ESM.docx]

**Additional file 3. Primer used for qRT-PCR**

| **Primer** | **Sequence (5′-3′)** | **Function** |
| --- | --- | --- |
| Q*fruI*-F | ACCATTCCTCCAGCCATTAC | A fragment of *fruI* ORF |
| Q*fruI*-R | GATATGGGTGGGCCTGTTAAT |  |
| Q*fruK*-F | GCTGATAAGCAAGAGCGTCTAA | A fragment of *fruK* ORF |
| Q*fruK*-R | ACCAGCTAGCCTTGGAAATAAG |  |
| Q*fruR*-F | GTTTAACCGCTCCTCCGATAA | A fragment of *fruR* ORF |
| Q*fruR*-R | TCTCCCTCACGAGCAATTAAC |  |
| Q*luxS*-F | TCCAGGATGAGGACATGAGCAC | A fragment of *luxS* ORF |
| Q*luxS*-R | GTAGCATCCTGTTTTCCCCACA |  |
| Q*ccpA*-F | GGTTCGTTTGGCAGGATACA | A fragment of *ccpA* ORF |
| Q*ccpA*-R | GCCTTCAGCATAACGGTAGTT |  |
| *recA*-F | ATTCAGGCGCAGTTGATTTAGTT | A fragment of *recA* |
| *recA* -R | TCAATCTCAGCACGAGGAACA |  |
