# Supplementary material for: luxS contributes to intramacrophage survival of Streptococcus agalactiae by positively affecting the expression of fruRKI operon
Source: Vet Res. 2023 Sep 27;54:83. doi: 10.1186/s13567-023-01210-9 (PMC10536698; doi:10.1186/s13567-023-01210-9)
Supplement: Supplementary file 4 — Additional file 4. The differentially expressed genes in ΔluxS compared with wild-type strain. [file 13567_2023_1210_MOESM4_ESM.docx]

**Additional file 4. The differentially expressed genes in Δ*luxS* compared with wild-type strain**

| **Number** | **locus_tag** | **Possible function** | **GenBank identification** | **Fold-change** | ***p*-value** |
| --- | --- | --- | --- | --- | --- |
| **Upregulated genes** |  |  |  |  |  |
| 1 | A964_RS08110 | kinase/pyrophosphorylase | WP_000390093.1 | 18.2563181 | 1.68E-35 |
| 2 | A964_RS08115 | helix-turn-helix transcriptional regulator | WP_071659924.1 | 15.4793694 | 6.55E-13 |
| 3 | A964_RS08105 | pyruvate%2C phosphate dikinase | WP_000448264.1 | 15.2117808 | 4.96E-38 |
| 4 | A964_RS08400 | MFS transporter | WP_000389617.1 | 6.5841403 | 1.47E-04 |
| 5 | A964_RS04880 | amino acid ABC transporter permease | WP_000120401.1 | 6.5456534 | 1.27E-36 |
| 6 | A964_RS08120 | 3-hydroxyacyl-CoA dehydrogenase | WP_000153116.1 | 6.25173942 | 7.68E-19 |
| 7 | A964_RS03230 | ABC transporter permease | WP_000594368.1 | 5.90116842 | 1.34E-52 |
| 8 | A964_RS07175 | DUF4044 domain-containing protein | WP_000863865.1 | 5.61744105 | 1.36E-02 |
| 9 | A964_RS04885 | amino acid ABC transporter ATP-binding protein | WP_000891276.1 | 5.18290132 | 3.11E-54 |
| 10 | A964_RS00145 | tRNA-Ala |  | 5.1389857 | 1.04E-02 |
| 11 | A964_RS00590 | tRNA-Ala |  | 5.1389857 | 1.04E-02 |
| 12 | A964_RS01055 | tRNA-Ala |  | 5.1389857 | 1.04E-02 |
| 13 | A964_RS01495 | tRNA-Ala |  | 5.1389857 | 1.04E-02 |
| 14 | A964_RS02025 | tRNA-Ala |  | 5.1389857 | 1.04E-02 |
| 15 | A964_RS02410 | tRNA-Ala |  | 5.1389857 | 1.04E-02 |
| 16 | A964_RS09845 | hypothetical protein | WP_001285708.1 | 5.08140402 | 2.67E-03 |
| 17 | A964_RS03240 | FtsX-like permease family protein | WP_000594351.1 | 5.06853561 | 1.37E-15 |
| 18 | A964_RS04890 | amino acid ABC transporter substrate-binding protein | WP_001873511.1 | 5.04062538 | 5.37E-40 |
| 19 | A964_RS02055 | enoyl-CoA hydratase | WP_000667368.1 | 5.00049938 | 7.45E-14 |
| 20 | A964_RS03235 | ABC transporter ATP-binding protein | WP_000353149.1 | 4.94280055 | 6.07E-28 |
| 21 | A964_RS03245 | response regulator transcription factor | WP_000699093.1 | 4.93103304 | 4.60E-09 |
| 22 | A964_RS00555 | adenylate kinase | WP_001050421.1 | 4.66763471 | 9.73E-28 |
| 23 | A964_RS02075 | enoyl-[acyl-carrier-protein] reductase FabK | WP_000857467.1 | 4.54458422 | 1.95E-19 |
| 24 | A964_RS00795 | dUTP diphosphatase | WP_000049186.1 | 4.48917779 | 2.88E-10 |
| 25 | A964_RS02060 | MarR family transcriptional regulator | WP_000455488.1 | 4.37125413 | 8.59E-26 |
| 26 | A964_RS09610 | phosphatase PAP2 family protein | WP_001036315.1 | 4.2485436 | 4.20E-04 |
| 27 | A964_RS00040 | RNA-binding S4 domain-containing protein | WP_001234967.1 | 4.23855972 | 8.07E-12 |
| 28 | A964_RS02080 | ACP S-malonyltransferase | WP_001044779.1 | 4.21274592 | 2.05E-29 |
| 29 | A964_RS02085 | 3-oxoacyl-[acyl-carrier-protein] reductase | WP_001176108.1 | 4.04848056 | 9.50E-27 |
| 30 | A964_RS02065 | ketoacyl-ACP synthase III | WP_000230695.1 | 4.02044501 | 5.31E-27 |
| 31 | A964_RS05005 | hypothetical protein | WP_001140791.1 | 3.9704757 | 3.10E-21 |
| 32 | A964_RS02070 | acyl carrier protein | WP_000257836.1 | 3.81122775 | 1.81E-20 |
| 33 | A964_RS06615 | KH domain-containing protein | WP_000379626.1 | 3.63347714 | 1.62E-42 |
| 34 | A964_RS10455 | hypothetical protein | WP_000027835.1 | 3.51442148 | 1.58E-02 |
| 35 | A964_RS04295 | glucosamine-6-phosphate deaminase | WP_001263956.1 | 3.46414601 | 3.76E-54 |
| 36 | A964_RS03740 | iron export ABC transporter permease subunit FetB | WP_000011926.1 | 3.43477323 | 1.92E-64 |
| 37 | A964_RS09440 | replication initiation factor domain-containing protein | WP_000483438.1 | 3.4295072 | 6.58E-03 |
| 38 | A964_RS03250 | HAMP domain-containing histidine kinase | WP_000734168.1 | 3.3666328 | 2.79E-16 |
| 39 | A964_RS03735 | ATP-binding cassette domain-containing protein | WP_000121094.1 | 3.33120898 | 7.13E-69 |
| 40 | A964_RS02090 | beta-ketoacyl-ACP synthase II | WP_000175070.1 | 3.28173946 | 2.16E-21 |
| 41 | A964_RS00545 | 50S ribosomal protein L15 | WP_000766093.1 | 3.17183996 | 1.85E-50 |
| 42 | A964_RS04895 | 30S ribosomal protein S20 | WP_001274006.1 | 3.14378936 | 3.06E-06 |
| 43 | A964_RS08100 | Asp-tRNA (Asn)/Glu-tRNA (Gln) amidotransferase subunit GatC | WP_000703032.1 | 3.12812077 | 1.86E-26 |
| 44 | A964_RS02120 | hypothetical protein | WP_000284171.1 | 3.05478667 | 5.29E-19 |
| 45 | A964_RS09670 | AzlC family ABC transporter permease | WP_000410672.1 | 3.0499711 | 1.11E-10 |
| 46 | A964_RS00140 | 16S ribosomal RNA |  | 3.02243123 | 3.07E-04 |
| 47 | A964_RS00530 | 50S ribosomal protein L18 | WP_001865622.1 | 2.98059013 | 2.98E-15 |
| 48 | A964_RS00540 | 50S ribosomal protein L30 | WP_000057245.1 | 2.97869749 | 1.17E-83 |
| 49 | A964_RS00535 | 30S ribosomal protein S5 | WP_000874200.1 | 2.97483842 | 2.64E-15 |
| 50 | A964_RS00585 | 16S ribosomal RNA |  | 2.96684777 | 2.45E-04 |
| 51 | A964_RS01490 | 16S ribosomal RNA |  | 2.96684777 | 2.45E-04 |
| 52 | A964_RS02405 | 16S ribosomal RNA |  | 2.96684777 | 2.45E-04 |
| 53 | A964_RS06620 | 30S ribosomal protein S16 | WP_000268757.1 | 2.9601627 | 5.18E-30 |
| 54 | A964_RS02020 | 16S ribosomal RNA |  | 2.91793591 | 1.40E-04 |
| 55 | A964_RS00505 | 50S ribosomal protein L24 | WP_000497687.1 | 2.88858079 | 1.25E-13 |
| 56 | A964_RS01110 | DNA-directed RNA polymerase subunit beta | WP_000907191.1 | 2.87821106 | 3.54E-39 |
| 57 | A964_RS00525 | 50S ribosomal protein L6 | WP_000086620.1 | 2.86953644 | 2.60E-13 |
| 58 | A964_RS02095 | acetyl-CoA carboxylase biotin carboxyl carrier protein | WP_000354298.1 | 2.86430854 | 7.38E-16 |
| 59 | A964_RS04385 | class 1b ribonucleoside-diphosphate reductase subunit alpha | WP_000053865.1 | 2.86333557 | 6.97E-13 |
| 60 | A964_RS00480 | 30S ribosomal protein S3 | WP_000529929.1 | 2.82892532 | 1.95E-50 |
| 61 | A964_RS00520 | 30S ribosomal protein S8 | WP_000245501.1 | 2.81896441 | 8.93E-60 |
| 62 | A964_RS02100 | 3-hydroxyacyl-ACP dehydratase FabZ | WP_000565430.1 | 2.80944274 | 1.03E-12 |
| 63 | A964_RS06990 | transporter substrate-binding domain-containing protein | WP_000037656.1 | 2.79674561 | 1.23E-15 |
| 64 | A964_RS00510 | 50S ribosomal protein L5 | WP_000013545.1 | 2.77871063 | 1.70E-47 |
| 65 | A964_RS00495 | 30S ribosomal protein S17 | WP_000440811.1 | 2.76432941 | 2.03E-12 |
| 66 | A964_RS00500 | 50S ribosomal protein L14 | WP_000615920.1 | 2.76385868 | 1.46E-99 |
| 67 | A964_RS00485 | 50S ribosomal protein L16 | WP_000960950.1 | 2.75833361 | 1.11E-62 |
| 68 | A964_RS00470 | 30S ribosomal protein S19 | WP_000533765.1 | 2.75606318 | 3.36E-84 |
| 69 | A964_RS00515 | type Z 30S ribosomal protein S14 | WP_001085698.1 | 2.74377949 | 8.52E-92 |
| 70 | A964_RS05530 | xanthine phosphoribosyltransferase | WP_000770389.1 | 2.73923522 | 1.05E-18 |
| 71 | A964_RS09995 | hypothetical protein | WP_001873352.1 | 2.73618874 | 9.59E-04 |
| 72 | A964_RS00580 | 50S ribosomal protein L17 | WP_000331497.1 | 2.72537653 | 5.17E-125 |
| 73 | A964_RS00550 | preprotein translocase subunit SecY | WP_000478891.1 | 2.71539409 | 1.95E-61 |
| 74 | A964_RS06580 | PTS transporter subunit IIC | WP_000185920.1 | 2.71519472 | 1.53E-55 |
| 75 | A964_RS10220 | B3/4 domain-containing protein | WP_000159986.1 | 2.70863767 | 1.16E-17 |
| 76 | A964_RS00475 | 50S ribosomal protein L22 | WP_000818141.1 | 2.69591668 | 1.94E-89 |
| 77 | A964_RS00460 | 50S ribosomal protein L23 | WP_001055343.1 | 2.68429169 | 1.56E-95 |
| 78 | A964_RS08665 | 30S ribosomal protein S12 | WP_001142328.1 | 2.68383274 | 3.76E-56 |
| 79 | A964_RS00465 | 50S ribosomal protein L2 | WP_000511737.1 | 2.67706556 | 6.27E-97 |
| 80 | A964_RS00570 | 30S ribosomal protein S11 | WP_001118387.1 | 2.66867134 | 6.61E-92 |
| 81 | A964_RS00490 | 50S ribosomal protein L29 | WP_000775731.1 | 2.6575167 | 8.27E-62 |
| 82 | A964_RS04470 | ATP-binding cassette domain-containing protein | WP_000953593.1 | 2.65531171 | 4.84E-02 |
| 83 | A964_RS00160 | tRNA-Val |  | 2.65227721 | 3.24E-02 |
| 84 | A964_RS03200 | 50S ribosomal protein L19 | WP_001068667.1 | 2.62983637 | 1.97E-36 |
| 85 | A964_RS00575 | DNA-directed RNA polymerase subunit alpha | WP_000568977.1 | 2.62775001 | 4.71E-74 |
| 86 | A964_RS02105 | acetyl-CoA carboxylase biotin carboxylase subunit | WP_000473511.1 | 2.62525116 | 3.16E-14 |
| 87 | A964_RS00455 | 50S ribosomal protein L4 | WP_000024418.1 | 2.61562617 | 1.87E-11 |
| 88 | A964_RS09625 | CPBP family intramembrane metalloprotease | WP_001071067.1 | 2.60678301 | 4.81E-06 |
| 89 | A964_RS06575 | 2-dehydropantoate 2-reductase | WP_000964162.1 | 2.58152698 | 1.71E-62 |
| 90 | A964_RS07720 | APC family permease | WP_000427463.1 | 2.57491286 | 1.24E-24 |
| 91 | A964_RS04435 | DUF3272 domain-containing protein | WP_000166926.1 | 2.56476328 | 4.12E-03 |
| 92 | A964_RS00450 | 50S ribosomal protein L3 | WP_000160205.1 | 2.54745149 | 7.25E-11 |
| 93 | A964_RS06150 | adenine phosphoribosyltransferase | WP_000365344.1 | 2.53377219 | 3.52E-52 |
| 94 | A964_RS05525 | purine permease | WP_000671152.1 | 2.51660285 | 4.78E-27 |
| 95 | A964_RS00560 | translation initiation factor IF-1 | WP_001040189.1 | 2.50505302 | 9.85E-11 |
| 96 | A964_RS00565 | 30S ribosomal protein S13 | WP_000090785.1 | 2.4982377 | 1.03E-97 |
| 97 | A964_RS10325 | 50S ribosomal protein L36 | WP_000868345.1 | 2.49738677 | 3.25E-51 |
| 98 | A964_RS00085 | 23S ribosomal RNA |  | 2.46210748 | 1.10E-03 |
| 99 | A964_RS00150 | 23S ribosomal RNA |  | 2.46210748 | 1.10E-03 |
| 100 | A964_RS00595 | 23S ribosomal RNA |  | 2.46210748 | 1.10E-03 |
| 101 | A964_RS01060 | 23S ribosomal RNA |  | 2.46210748 | 1.10E-03 |
| 102 | A964_RS01500 | 23S ribosomal RNA |  | 2.46210748 | 1.10E-03 |
| 103 | A964_RS02030 | 23S ribosomal RNA |  | 2.46210748 | 1.10E-03 |
| 104 | A964_RS02415 | 23S ribosomal RNA |  | 2.46210748 | 1.10E-03 |
| 105 | A964_RS00445 | 30S ribosomal protein S10 | WP_001284518.1 | 2.46187399 | 2.08E-42 |
| 106 | A964_RS01115 | DNA-directed RNA polymerase subunit beta' | WP_000228729.1 | 2.454656 | 1.09E-47 |
| 107 | A964_RS02050 | HAD family phosphatase | WP_000335411.1 | 2.4451136 | 1.68E-02 |
| 108 | A964_RS02110 | acetyl-CoA carboxylase carboxyltransferase subunit beta | WP_001173385.1 | 2.44189185 | 1.27E-12 |
| 109 | A964_RS00105 | tRNA-Lys |  | 2.43530255 | 9.58E-03 |
| 110 | A964_RS10095 | 30S ribosomal protein S4 | WP_000092759.1 | 2.43396635 | 1.29E-30 |
| 111 | A964_RS02115 | acetyl-CoA carboxylase carboxyl transferase subunit alpha | WP_001058274.1 | 2.40633483 | 1.27E-11 |
| 112 | A964_RS00100 | tRNA-Asp |  | 2.37008407 | 9.54E-03 |
| 113 | A964_RS04380 | class 1b ribonucleoside-diphosphate reductase subunit beta | WP_000214845.1 | 2.36882562 | 1.27E-77 |
| 114 | A964_RS08660 | 30S ribosomal protein S7 | WP_000087842.1 | 2.32967063 | 1.40E-70 |
| 115 | A964_RS07950 | branched-chain amino acid transport system II carrier protein | WP_000769345.1 | 2.32894151 | 6.36E-12 |
| 116 | A964_RS07725 | Trk system potassium transporter TrkA | WP_001220338.1 | 2.30685641 | 4.31E-50 |
| 117 | A964_RS03170 | type B 50S ribosomal protein L31 | WP_000710758.1 | 2.30502337 | 1.26E-79 |
| 118 | A964_RS05845 | sodium:alanine symporter family protein | WP_000956723.1 | 2.28101305 | 3.18E-02 |
| 119 | A964_RS09665 | branched-chain amino acid transporter | WP_000610955.1 | 2.27893213 | 7.23E-03 |
| 120 | A964_RS03760 | D-lactate dehydrogenase | WP_000770077.1 | 2.2714273 | 3.98E-77 |
| 121 | A964_RS05035 | tyrosine recombinase XerS | WP_000817930.1 | 2.25977867 | 4.42E-49 |
| 122 | A964_RS01295 | 30S ribosomal protein S15 | WP_001018249.1 | 2.25737305 | 1.56E-07 |
| 123 | A964_RS05060 | putative DNA-binding protein | WP_000402075.1 | 2.24924027 | 6.14E-14 |
| 124 | A964_RS01760 | 16S rRNA (cytosine(1402)-N(4))-methyltransferase RsmH | WP_000180266.1 | 2.23644873 | 9.65E-22 |
| 125 | A964_RS03755 | PTS transporter subunit IIC | WP_001286423.1 | 2.22671196 | 5.58E-54 |
| 126 | A964_RS00170 | tRNA-Lys |  | 2.22496971 | 9.12E-03 |
| 127 | A964_RS01075 | 4-(cytidine 5'-diphospho)-2-C-methyl-D-erythritol kinase | WP_000688172.1 | 2.21243812 | 4.70E-49 |
| 128 | A964_RS00050 | hypothetical protein | WP_001061058.1 | 2.18728314 | 3.98E-04 |
| 129 | A964_RS00045 | septum formation initiator family protein | WP_000042845.1 | 2.17858068 | 5.48E-06 |
| 130 | A964_RS04290 | VIT family protein | WP_000656582.1 | 2.17387945 | 5.24E-17 |
| 131 | A964_RS08095 | Asp-Trna (Asn)/Glu-tRNA (Gln) amidotransferase subunit GatA | WP_000009529.1 | 2.16453181 | 1.79E-70 |
| 132 | A964_RS08590 | aminotransferase class V-fold PLP-dependent enzyme | WP_000902193.1 | 2.16176776 | 1.51E-04 |
| 133 | A964_RS03765 | MFS transporter | WP_000416102.1 | 2.15677598 | 1.56E-02 |
| 134 | A964_RS01800 | DUF4059 family protein | WP_000478229.1 | 2.15162059 | 2.90E-04 |
| 135 | A964_RS08345 | ATP-dependent RecD-like DNA helicase | WP_000451609.1 | 2.14926449 | 1.54E-37 |
| 136 | A964_RS08975 | alkyl hydroperoxide reductase subunit F | WP_000242129.1 | 2.14832611 | 2.05E-68 |
| 137 | A964_RS08970 | peroxiredoxin | WP_000060538.1 | 2.14316092 | 4.30E-22 |
| 138 | A964_RS00165 | tRNA-Asp |  | 2.13266813 | 1.65E-02 |
| 139 | A964_RS07430 | IS3-like element IS861 family transposase | WP_088181631.1 | 2.12885792 | 4.10E-02 |
| 140 | A964_RS07985 | gamma-glutamyl-gamma-aminobutyrate hydrolase family protein | WP_000956420.1 | 2.12827238 | 4.26E-18 |
| 141 | A964_RS06145 | DnaD domain-containing protein | WP_000221659.1 | 2.11345761 | 5.97E-17 |
| 142 | A964_RS02510 | IS3-like element IS861 family transposase | WP_088181631.1 | 2.08443336 | 4.09E-02 |
| 143 | A964_RS01835 | DUF1273 domain-containing protein | WP_000843096.1 | 2.07944283 | 1.40E-02 |
| 144 | A964_RS09055 | glycoside hydrolase family 73 protein | WP_000842266.1 | 2.07608829 | 2.03E-05 |
| 145 | A964_RS08810 | transcription antiterminator | WP_000584678.1 | 2.04986155 | 5.58E-05 |
| 146 | A964_RS09990 | hypothetical protein | WP_000614740.1 | 2.04594964 | 2.68E-02 |
| 147 | A964_RS04030 | bifunctional diaminohydroxyphosphoribosylaminopyrimidine deaminase/5-amino-6-(5-phosphoribosylamino) uracil reductase RibD | WP_000975998.1 | 2.04102029 | 1.86E-04 |
| 148 | A964_RS07390 | 50S ribosomal protein L1 | WP_001085664.1 | 2.04083884 | 3.85E-27 |
| 149 | A964_RS03695 | ABC transporter permease | WP_001055322.1 | 2.03902216 | 2.19E-02 |
| 150 | A964_RS08780 | 50S ribosomal protein L34 | WP_000831903.1 | 2.03493465 | 7.02E-03 |
| 151 | A964_RS00785 | alpha/beta hydrolase | WP_000723856.1 | 2.03056235 | 9.59E-09 |
| 152 | A964_RS06980 | 30S ribosomal protein S21 | WP_000048058.1 | 2.02918083 | 2.51E-02 |
| 153 | A964_RS03190 | chorismate mutase | WP_000418127.1 | 2.02457183 | 2.32E-15 |
| 154 | A964_RS10030 | YfcC family protein | WP_001114291.1 | 2.00618952 | 2.71E-32 |
| 155 | A964_RS07415 | LysR family transcriptional regulator | WP_000280435.1 | 2.00288641 | 2.00E-04 |
| **Downregulated genes** |  |  |  |  |  |
| 1 | A964_RS04240 | PRD domain-containing protein | WP_000584608.1 | 12.2833452 | 4.98E-83 |
| 2 | A964_RS00290 | amidophosphoribosyltransferase | WP_000220672.1 | 11.7025231 | 1.45E-31 |
| 3 | A964_RS04255 | glycoside hydrolase family 1 protein | WP_000215675.1 | 10.2165233 | 2.02E-26 |
| 4 | A964_RS00305 | GNAT family N-acetyltransferase | WP_000780020.1 | 9.14596727 | 9.41E-25 |
| 5 | A964_RS00295 | phosphoribosylformylglycinamidine cyclo-ligase | WP_001291325.1 | 8.22649669 | 1.27E-21 |
| 6 | A964_RS00300 | phosphoribosylglycinamide formyltransferase | WP_000685111.1 | 8.05588867 | 5.27E-68 |
| 7 | A964_RS00285 | phosphoribosylformylglycinamidine synthase | WP_001042263.1 | 7.5327171 | 1.50E-09 |
| 8 | A964_RS05330 | carbamoyl-phosphate synthase large subunit | WP_001126458.1 | 6.02006171 | 1.08E-22 |
| 9 | A964_RS03640 | hypothetical protein | WP_000033001.1 | 5.65557258 | 2.40E-19 |
| 10 | A964_RS03635 | hypothetical protein | WP_000118223.1 | 5.50521575 | 7.75E-95 |
| 11 | A964_RS03630 | aminomethyl transferase family protein | WP_001092618.1 | 5.50429727 | 2.08E-28 |
| 12 | A964_RS09720 | YSIRK signal domain/LPXTG anchor domain surface protein | WP_001063288.1 | 5.50205736 | 1.37E-17 |
| 13 | A964_RS03645 | hypothetical protein | WP_001068960.1 | 5.49468599 | 6.28E-94 |
| 14 | A964_RS05335 | glutamine-hydrolyzing carbamoyl-phosphate synthase small subunit | WP_000826107.1 | 5.4048327 | 3.46E-58 |
| 15 | A964_RS05835 | PaaI family thioesterase | WP_000671265.1 | 5.16988687 | 1.53E-05 |
| 16 | A964_RS05340 | aspartate carbamoyltransferase catalytic subunit | WP_001016473.1 | 5.03885629 | 8.23E-31 |
| 17 | A964_RS00280 | phosphoribosylaminoimidazolesuccinocarboxamide synthase | WP_000184493.1 | 4.87207959 | 8.81E-05 |
| 18 | A964_RS03625 | hypothetical protein | WP_000650746.1 | 4.83823195 | 6.18E-26 |
| 19 | A964_RS06200 | pullulanase | WP_000823021.1 | 4.70978146 | 7.98E-18 |
| 20 | A964_RS05320 | hypothetical protein | WP_001871005.1 | 4.24663712 | 1.43E-73 |
| 21 | A964_RS03620 | ABC transporter permease | WP_000462408.1 | 4.21535906 | 4.30E-74 |
| 22 | A964_RS06570 | YSIRK-type signal peptide-containing protein | WP_000976031.1 | 4.10605399 | 6.77E-21 |
| 23 | A964_RS00310 | bifunctional phosphoribosylaminoimidazolecarboxamide formyltransferase/IMP cyclohydrolase | WP_000166558.1 | 4.10509724 | 6.04E-15 |
| 24 | A964_RS04265 | glycerate kinase | WP_000869908.1 | 3.94038449 | 4.09E-13 |
| 25 | A964_RS03615 | ABC transporter ATP-binding protein | WP_000403526.1 | 3.78569791 | 2.00E-15 |
| 26 | A964_RS06550 | PTS transporter subunit EIIA | WP_000701403.1 | 3.72573614 | 2.13E-23 |
| 27 | A964_RS06485 | bifunctional metallophosphatase/5'-nucleotidase | WP_000726930.1 | 3.59710767 | 3.88E-13 |
| 28 | A964_RS03610 | beta-hydroxyacyl-ACP dehydratase | WP_000164166.1 | 3.55602431 | 1.99E-91 |
| 29 | A964_RS07010 | hypothetical protein | WP_000220571.1 | 3.54408309 | 6.50E-08 |
| 30 | A964_RS05325 | hypothetical protein | WP_000921237.1 | 3.49306472 | 1.21E-32 |
| 31 | A964_RS03650 | hypothetical protein | WP_001288731.1 | 3.43250958 | 4.09E-02 |
| 32 | A964_RS03605 | acyl carrier protein | WP_000611493.1 | 3.34624579 | 9.83E-56 |
| 33 | A964_RS04270 | GntP family permease | WP_000388411.1 | 3.3404671 | 7.28E-13 |
| 34 | A964_RS01405 | hypothetical protein | WP_000130119.1 | 3.23800248 | 7.59E-04 |
| 35 | A964_RS01095 | hypothetical protein | WP_000777427.1 | 3.18948666 | 9.30E-09 |
| 36 | A964_RS03600 | SDR family oxidoreductase | WP_000861303.1 | 3.02931928 | 7.80E-49 |
| 37 | A964_RS00365 | alpha/beta fold hydrolase | WP_000573443.1 | 2.9752137 | 3.32E-15 |
| 38 | A964_RS06555 | 1-phosphofructokinase | WP_000640758.1 | 2.96067443 | 1.60E-11 |
| 39 | A964_RS08850 | fructose-6-phosphate aldolase | WP_000873297.1 | 2.94628409 | 1.20E-65 |
| 40 | A964_RS00360 | ROK family protein | WP_000198870.1 | 2.92536889 | 1.37E-13 |
| 41 | A964_RS00325 | N-acetylmannosamine-6-phosphate 2-epimerase | WP_001120327.1 | 2.91142054 | 1.47E-34 |
| 42 | A964_RS00350 | YesL family protein | WP_000710135.1 | 2.9113514 | 8.39E-11 |
| 43 | A964_RS00355 | N-acetylneuraminate lyase | WP_000654092.1 | 2.89710698 | 2.97E-12 |
| 44 | A964_RS09190 | pneumococcal-type histidine triad protein | WP_001080364.1 | 2.86770745 | 3.62E-07 |
| 45 | A964_RS03800 | SDR family oxidoreductase | WP_000034790.1 | 2.85004784 | 2.36E-47 |
| 46 | A964_RS06545 | DUF2127 domain-containing protein | WP_000598909.1 | 2.84310503 | 2.37E-14 |
| 47 | A964_RS08855 | L-ribulose-5-phosphate 4-epimerase | WP_001129979.1 | 2.81598324 | 2.12E-25 |
| 48 | A964_RS02625 | PTS sugar transporter subunit IIB | WP_000734774.1 | 2.79435068 | 6.62E-67 |
| 49 | A964_RS08725 | hypothetical protein | WP_000263623.1 | 2.77930113 | 3.82E-05 |
| 50 | A964_RS00335 | sugar ABC transporter permease | WP_001260027.1 | 2.77325247 | 1.36E-10 |
| 51 | A964_RS00340 | carbohydrate ABC transporter permease | WP_000730582.1 | 2.74722775 | 1.72E-11 |
| 52 | A964_RS00330 | carbohydrate ABC transporter substrate-binding protein | WP_000023471.1 | 2.74240873 | 2.47E-13 |
| 53 | A964_RS08865 | 3-keto-L-gulonate-6-phosphate decarboxylase UlaD | WP_000166859.1 | 2.71100901 | 4.26E-44 |
| 54 | A964_RS00345 | DUF386 family protein | WP_000638660.1 | 2.70875989 | 2.89E-56 |
| 55 | A964_RS03660 | S8 family serine peptidase | WP_000165742.1 | 2.69355184 | 4.22E-08 |
| 56 | A964_RS08860 | L-ribulose-5-phosphate 3-epimerase | WP_000197816.1 | 2.61933835 | 5.67E-56 |
| 57 | A964_RS03305 | endonuclease | WP_000823925.1 | 2.58150359 | 2.68E-33 |
| 58 | A964_RS03810 | beta-hexosamidase | WP_000149447.1 | 2.55286202 | 5.75E-27 |
| 59 | A964_RS08470 | transketolase | WP_000141745.1 | 2.54221359 | 4.27E-45 |
| 60 | A964_RS05345 | dihydroorotase | WP_000275473.1 | 2.50074271 | 5.23E-21 |
| 61 | A964_RS09275 | ABC transporter permease subunit | WP_079219497.1 | 2.48821337 | 2.97E-29 |
| 62 | A964_RS03665 | hypothetical protein | WP_079262263.1 | 2.47411329 | 1.54E-09 |
| 63 | A964_RS06560 | DeoR/GlpR transcriptional regulator | WP_000920253.1 | 2.4728028 | 3.35E-24 |
| 64 | A964_RS09280 | hypothetical protein | WP_001870658.1 | 2.44291171 | 1.41E-15 |
| 65 | A964_RS08870 | PTS transporter subunit EIIA | WP_001049922.1 | 2.44131281 | 7.34E-29 |
| 66 | A964_RS02560 | YSIRK signal domain/LPXTG anchor domain surface protein | WP_000489957.1 | 2.44050995 | 7.31E-41 |
| 67 | A964_RS05295 | hypothetical protein | WP_001091721.1 | 2.43952621 | 1.50E-13 |
| 68 | A964_RS03595 | hypothetical protein | WP_000859501.1 | 2.43852538 | 1.19E-39 |
| 69 | A964_RS00845 | ribokinase | WP_000065857.1 | 2.43366961 | 5.15E-38 |
| 70 | A964_RS03805 | HAD family hydrolase | WP_000228631.1 | 2.4265187 | 3.51E-10 |
| 71 | A964_RS02620 | PTS galactitol transporter subunit IIC | WP_000884278.1 | 2.42219194 | 1.08E-10 |
| 72 | A964_RS00835 | sugar ABC transporter ATP-binding protein | WP_000687215.1 | 2.41984352 | 1.67E-84 |
| 73 | A964_RS00880 | argininosuccinate lyase | WP_000164574.1 | 2.40649759 | 3.60E-44 |
| 74 | A964_RS08480 | ribulose-phosphate 3-epimerase | WP_001174252.1 | 2.40424519 | 2.71E-18 |
| 75 | A964_RS00840 | D-ribose pyranase | WP_000750739.1 | 2.40362033 | 1.26E-32 |
| 76 | A964_RS08010 | dihydroxyacetone kinase subunit L | WP_000453282.1 | 2.40312562 | 4.60E-91 |
| 77 | A964_RS08475 | fructose-6-phosphate aldolase | WP_000667769.1 | 2.39590926 | 1.23E-38 |
| 78 | A964_RS08015 | PTS-dependent dihydroxyacetone kinase phosphotransferase subunit DhaM | WP_001279899.1 | 2.38889524 | 2.25E-96 |
| 79 | A964_RS03795 | mannonate dehydratase | WP_000426046.1 | 2.35729168 | 2.04E-27 |
| 80 | A964_RS03130 | ECF transporter S component | WP_000167491.1 | 2.35149132 | 6.26E-04 |
| 81 | A964_RS05840 | uracil transporter | WP_000174084.1 | 2.34661245 | 1.16E-08 |
| 82 | A964_RS08645 | 5'-nucleotidase%2C lipoprotein e(P4) family | WP_000858929.1 | 2.33969223 | 7.52E-59 |
| 83 | A964_RS02635 | sugar ABC transporter substrate-binding protein | WP_000738091.1 | 2.32376861 | 9.34E-39 |
| 84 | A964_RS08020 | aquaporin family protein | WP_000191476.1 | 2.30055144 | 1.09E-95 |
| 85 | A964_RS03135 | hypothetical protein | WP_001031100.1 | 2.24583874 | 3.88E-07 |
| 86 | A964_RS08005 | dihydroxyacetone kinase subunit DhaK | WP_000722647.1 | 2.23408666 | 5.40E-69 |
| 87 | A964_RS05220 | carbon starvation protein A | WP_000256021.1 | 2.22310038 | 8.85E-49 |
| 88 | A964_RS01440 | hypothetical protein | WP_000259069.1 | 2.20732441 | 2.39E-11 |
| 89 | A964_RS07550 | hypothetical protein | WP_000760433.1 | 2.20104315 | 5.07E-05 |
| 90 | A964_RS01290 | oxidoreductase | WP_001203071.1 | 2.1876102 | 1.32E-26 |
| 91 | A964_RS08730 | hypothetical protein | WP_001173857.1 | 2.15014732 | 1.28E-08 |
| 92 | A964_RS08875 | PTS sugar transporter subunit IIB | WP_000241475.1 | 2.12676362 | 7.80E-08 |
| 93 | A964_RS02265 | alpha/beta hydrolase | WP_000804611.1 | 2.09872006 | 4.75E-08 |
| 94 | A964_RS07275 | hypothetical protein | WP_000039430.1 | 2.09835121 | 6.97E-03 |
| 95 | A964_RS00830 | hypothetical protein | WP_000978822.1 | 2.09798378 | 8.38E-63 |
| 96 | A964_RS06345 | ATP-dependent Clp protease ATP-binding subunit | WP_000002804.1 | 2.09580368 | 2.23E-60 |
| 97 | A964_RS05215 | L%2CD-transpeptidase | WP_001231795.1 | 2.0910734 | 1.93E-02 |
| 98 | A964_RS08485 | PTS fructose transporter subunit IIC | WP_000654240.1 | 2.08257998 | 3.38E-21 |
| 99 | A964_RS05350 | orotate phosphoribosyltransferase | WP_000362326.1 | 2.07132525 | 7.05E-05 |
| 100 | A964_RS05290 | type VII secretion protein EssB | WP_000447063.1 | 2.06590255 | 5.78E-44 |
| 101 | A964_RS08880 | PTS sugar transporter subunit IIC | WP_000427087.1 | 2.04206391 | 1.40E-60 |
| 102 | A964_RS05300 | type VII secretion protein EssA | WP_000769471.1 | 2.04151531 | 1.94E-18 |
| 103 | A964_RS00425 | bifunctional acetaldehyde-CoA/alcohol dehydrogenase | WP_000137036.1 | 2.04120354 | 1.86E-80 |
| 104 | A964_RS03790 | glucuronate isomerase | WP_000143795.1 | 2.03874974 | 8.19E-35 |
| 105 | A964_RS03330 | ClbS/DfsB family four-helix bundle protein | WP_001261516.1 | 2.03427938 | 7.64E-06 |
| 106 | A964_RS08735 | DUF2974 domain-containing protein | WP_000416817.1 | 2.02812677 | 4.84E-19 |
| 107 | A964_RS06975 | large conductance mechanosensitive channel protein MscL | WP_000588846.1 | 2.0154608 | 1.41E-51 |
| 108 | A964_RS06430 | DUF4649 family protein | WP_000594205.1 | 2.00958722 | 1.37E-02 |
| 109 | A964_RS07910 | LemA family protein | WP_000537055.1 | 2.00316616 | 9.12E-50 |
