# Supplementary material for: luxS contributes to intramacrophage survival of Streptococcus agalactiae by positively affecting the expression of fruRKI operon
Source: Vet Res. 2023 Sep 27;54:83. doi: 10.1186/s13567-023-01210-9 (PMC10536698; doi:10.1186/s13567-023-01210-9)
Supplement: Supplementary file 5 — Additional file 5. The GO enrichment analysis of down-regulated gene in ΔluxS compared with wild-type strain. [file 13567_2023_1210_MOESM5_ESM.docx]

**Additional file 5 The GO enrichment analysis of down-regulated gene in Δ*luxS* compared with wild-type strain**

| Gene Ontology ID | term | Enrichment score | *P*-value | Gene |
| --- | --- | --- | --- | --- |
| GO:0006189 | de novo' IMP biosynthetic process | 10.51851852 | 1.44E-05 | A964_RS00280; A964_RS00285; A964_RS00290; A964_RS00295 |
| GO:0009401 | phosphoenolpyruvate-dependent sugar phosphotransferase system | 4.974974975 | 4.25E-05 | A964_RS02620; A964_RS06550; A964_RS08015; A964_RS08485; A964_RS08870; A964_RS08875; A964_RS08880 |
| GO:0019854 | L-ascorbic acid catabolic process | 17.5308642 | 5.21E-05 | A964_RS08860; A964_RS08865 |
| GO:0019262 | N-acetylneuraminate catabolic process | 13.14814815 | 2.03E-04 | A964_RS00325; A964_RS00355 |
| GO:0044205 | 'de novo' UMP biosynthetic process | 8.765432099 | 2.05E-04 | A964_RS05340; A964_RS05345; A964_RS05350 |
| GO:0006207 | 'de novo' pyrimidine nucleobase biosynthetic process | 8.765432099 | 9.60E-04 | A964_RS05340; A964_RS08865 |
| GO:0006633 | fatty acid biosynthetic process | 5.634920635 | 1.41E-03 | A964_RS03600; A964_RS03610; A964_RS03635 |
| GO:0005975 | carbohydrate metabolic process | 3.627075351 | 3.90E-03 | A964_RS00325; A964_RS00355; A964_RS04255; A964_RS06200 |
| GO:0055085 | transmembrane transport | 3.155555556 | 1.30E-02 | A964_RS00335; A964_RS00340; A964_RS02635 |
| GO:0005618 | cell wall | 5.259259259 | 1.87E-03 | A964_RS02560; A964_RS03660; A964_RS06485 |
| GO:0005576 | extracellular region | 3.756613757 | 3.32E-03 | A964_RS02560; A964_RS03660; A964_RS05215; A964_RS06485 |
| GO:0016020 | membrane | 4.781144781 | 6.91E-03 | A964_RS02560; A964_RS03660 |
| GO:0005887 | integral component of plasma membrane | 1.46090535 | 1.54E-01 | A964_RS04270; A964_RS05220 |
| GO:0016021 | integral component of membrane | 1.047661207 | 3.52E-01 | A964_RS00335; A964_RS00340; A964_RS02620; A964_RS05840; A964_RS06550; A964_RS07910; A964_RS08015; A964_RS08020; A964_RS08485; A964_RS08880 |
| GO:0005886 | plasma membrane | 0.950468541 | 5.06E-01 | A964_RS00335; A964_RS00340; A964_RS00835; A964_RS02620; A964_RS03615; A964_RS05215; A964_RS05840; A964_RS06550; A964_RS07910; A964_RS08020; A964_RS08485; A964_RS08880 |
| GO:0005737 | cytoplasm | 0.552774997 | 9.60E-01 | A964_RS00285; A964_RS00295; A964_RS00355; A964_RS00365; A964_RS03610; A964_RS08870; A964_RS08875 |
| GO:0005351 | carbohydrate:proton symporter activity | 17.5308642 | 5.21E-05 | A964_RS06550; A964_RS08485 |
| GO:0022877 | protein-N(PI)-phosphohistidine-fructose phosphotransferase system transporter activity | 13.14814815 | 2.03E-04 | A964_RS06550; A964_RS08485 |
| GO:0016301 | kinase activity | 2.544802867 | 2.74E-02 | A964_RS08485; A964_RS08870; A964_RS08875 |
| GO:0000287 | magnesium ion binding | 1.878306878 | 5.76E-02 | A964_RS00285; A964_RS00290; A964_RS05350; A964_RS08865 |
| GO:0008270 | zinc ion binding | 1.16872428 | 2.43E-01 | A964_RS05345; A964_RS08855 |
| GO:0005524 | ATP binding | 0.72541507 | 8.06E-01 | A964_RS00280; A964_RS00285; A964_RS00295; A964_RS00835; A964_RS03615; A964_RS04265; A964_RS06345; A964_RS08015 |
| GO:0046872 | metal ion binding | 0.502476999 | 8.67E-01 | A964_RS01290; A964_RS06485; A964_RS08480 |
