# Supplementary figures and images for: luxS contributes to intramacrophage survival of Streptococcus agalactiae by positively affecting the expression of fruRKI operon
Source: Vet Res. 2023 Sep 27;54:83. doi: 10.1186/s13567-023-01210-9 (PMC10536698; doi:10.1186/s13567-023-01210-9)

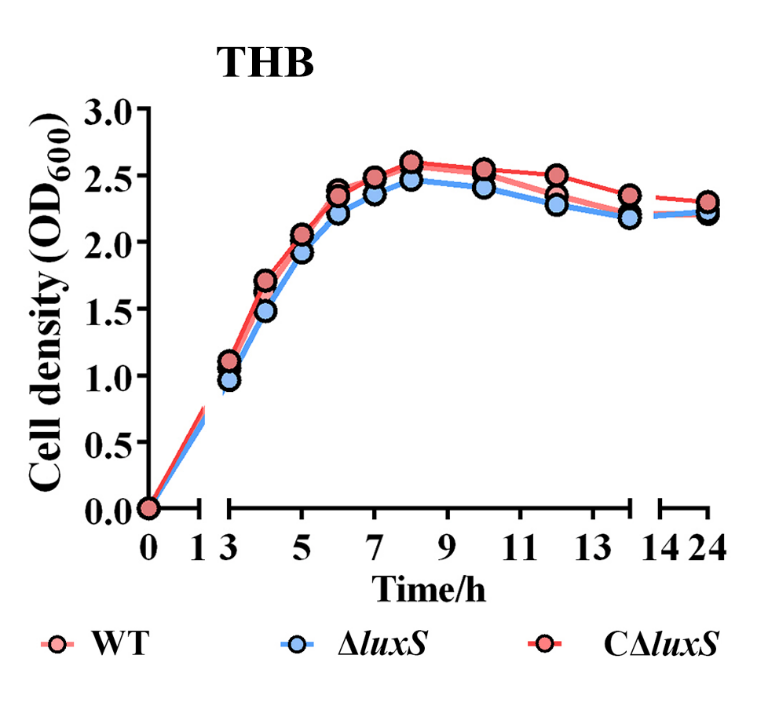

Supplement: Supplementary file 6 — Additional file 6. The growth of the WT, ΔluxS and CΔluxS strains in THB. [file 13567_2023_1210_MOESM6_ESM.docx]

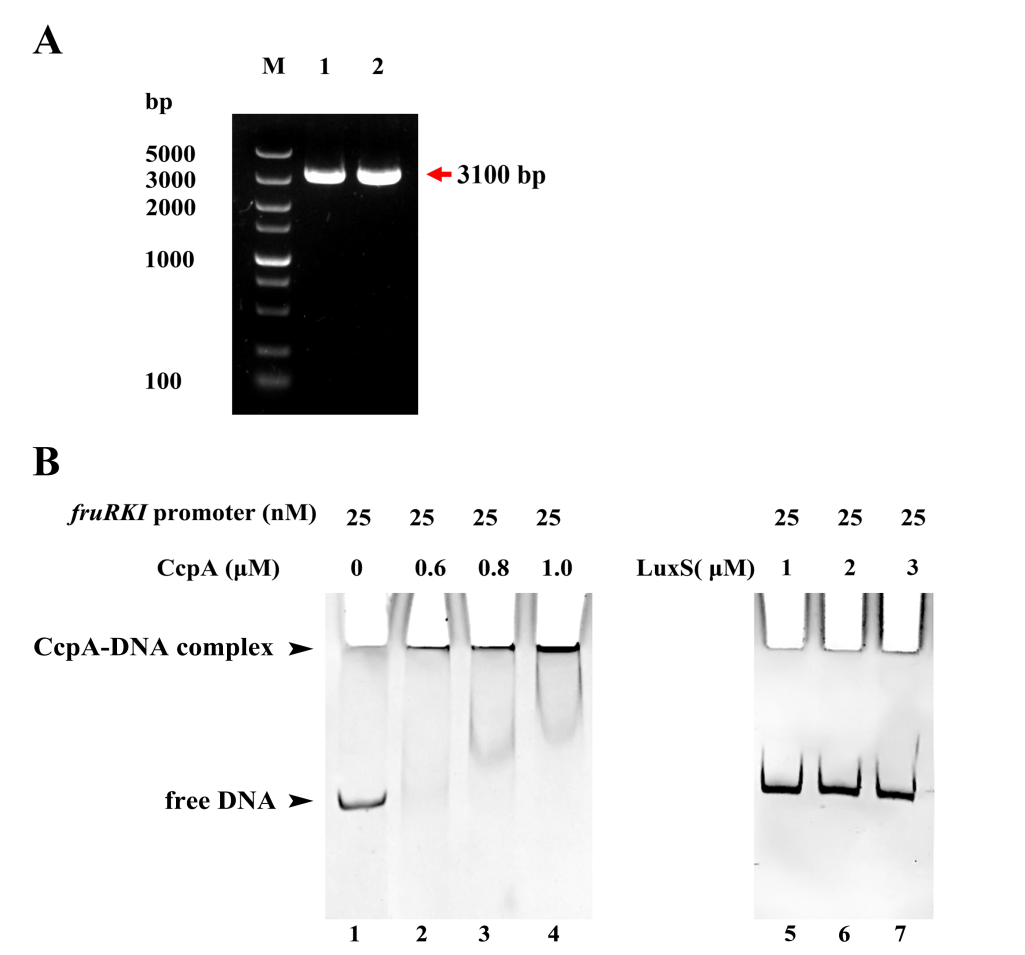

Supplement: Supplementary file 7 — Additional file 7. LuxS protein could not bind to the promoter of fruRKI operon. (A) The fruRKI operon was identified in the genome of S. agalactiae GD201008-001. Lane 1. A fragment amplified by PCR from the cDNA obtained by reverse trancription. Lane 2. A fragment amplified by PCR from genomic DNA as the positive control. M. DNA marker. (B) Binding ability of LuxS protein to the fruRKI promoter. Lane 1. Negative control (25 nM of fruRKI promoter). Lane 2–4. Positive controls. Binding reaction to 25 nM of fruRKI promoter with CcpA protein at a range of concentrations from 0.6 to 1 μM. Lane 5–7. Binding reaction to 50 nM of fruRKI promoter with LuxS protein at a range of concentrations from 1 to 3 μM. [file 13567_2023_1210_MOESM7_ESM.docx]

**Additional file 8 Relative mRNA levels of *ccpA* gene by real-time PCR.**


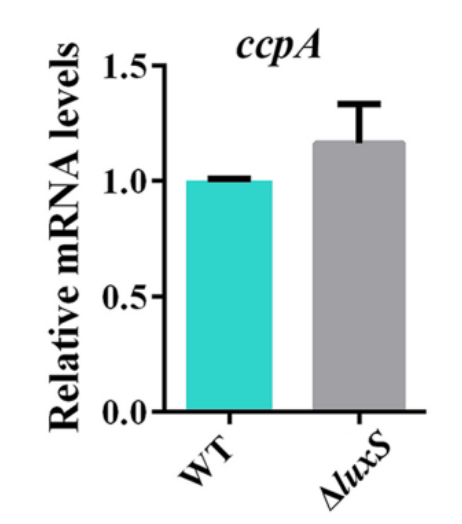

Supplement: Supplementary file 8 — Additional file 8. Relative mRNA levels of ccpA gene by real-time PCR. [file 13567_2023_1210_MOESM8_ESM.docx]

**Additional file** **9** **The model for the the regulation between luxS-fruRKI-CcpA.**


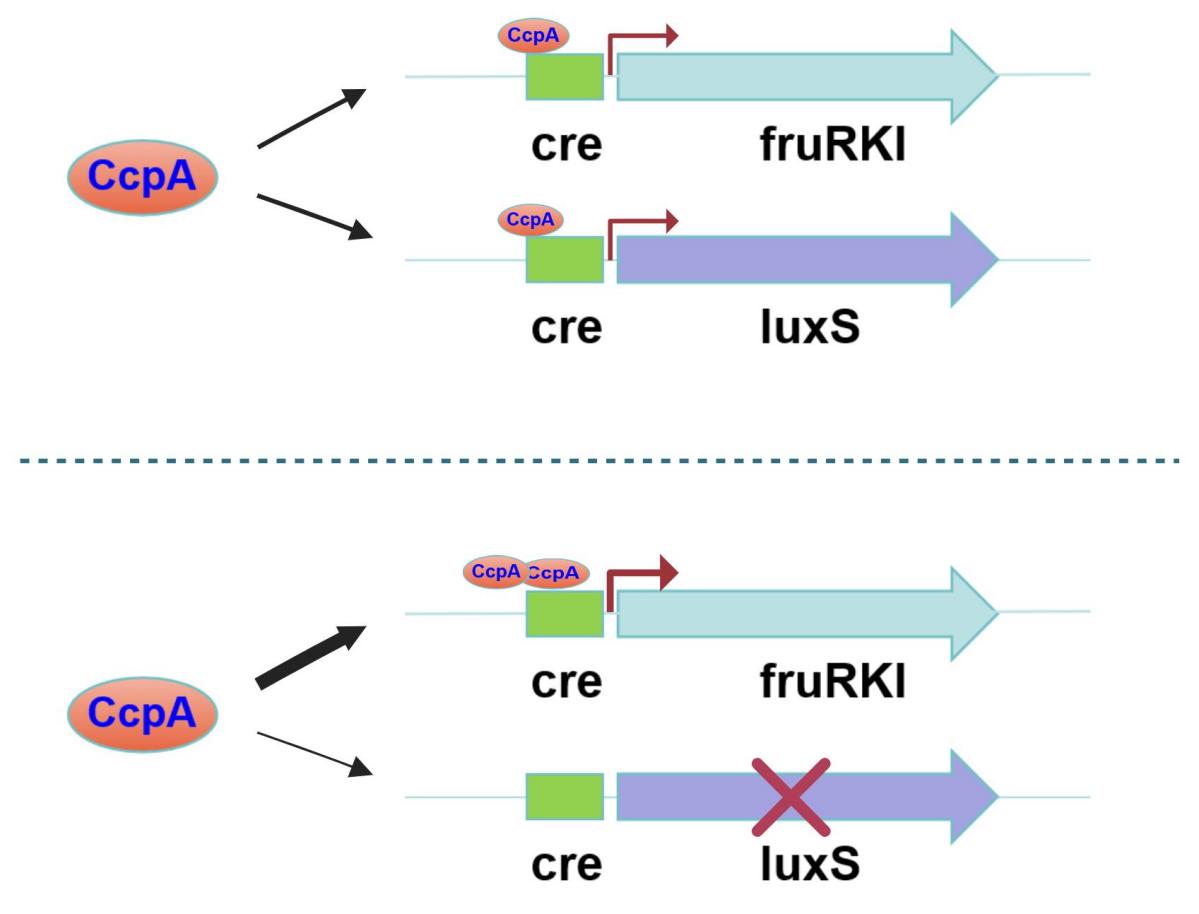

Supplement: Supplementary file 9 — Additional file 9. The model for the the regulation between luxS-fruRKI-CcpA. [file 13567_2023_1210_MOESM9_ESM.docx]

**Additional file** **11** **Competitive EMSA analyses the binding of CcpA to *ptsG* (A) or *rbsR* (B) promoters**


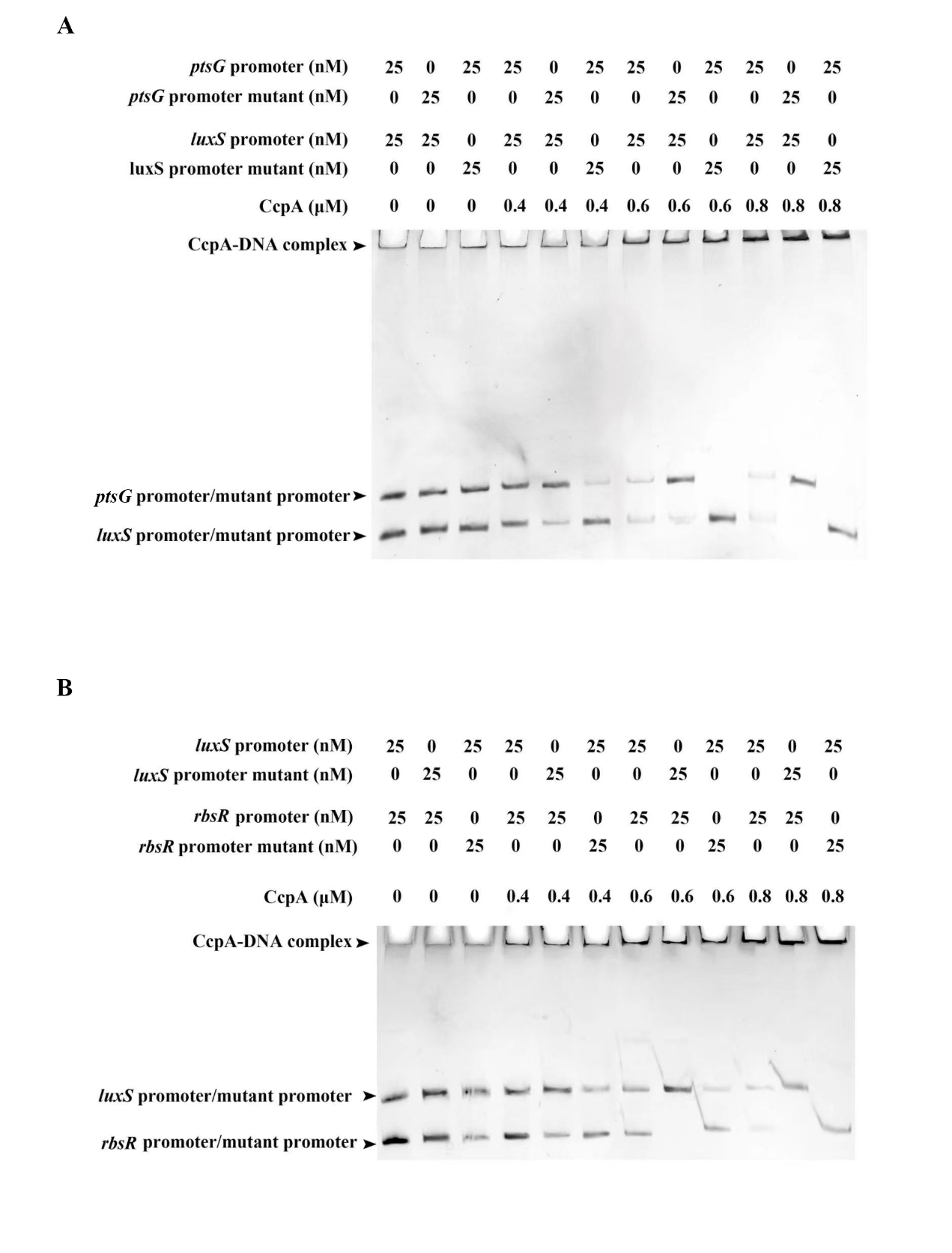

Supplement: Supplementary file 11 — Additional file 11. Competitive EMSA analyses the binding of CcpA to ptsG (A) or rbsR (B) promoters. [file 13567_2023_1210_MOESM11_ESM.docx]
