# Supplementary material for: luxS contributes to intramacrophage survival of Streptococcus agalactiae by positively affecting the expression of fruRKI operon
Source: Vet Res. 2023 Sep 27;54:83. doi: 10.1186/s13567-023-01210-9 (PMC10536698; doi:10.1186/s13567-023-01210-9)
Supplement: Supplementary file 10 — Additional file 10. The cre regions in the promoters of the downregulated genes in RNA-seq. [file 13567_2023_1210_MOESM10_ESM.docx]

| **NO.** | **Gene** | **Locus tag** | ***cre* sites** |
| --- | --- | --- | --- |
| 1 | *bglP* | A964_RS04250 | ATTAAGACGTTTTCAG |
| 2 | *bglH* | A964_RS04255 | ATTAAGACGTTTTCAG |
| 3 | *rbsB* | A964_RS00825 | TAGTAAGCGATTACAA |
| 4 | *rbsK* | A964_RS00830 | TAGTAAGCGATTACAA |
| 5 | *rbsD* | A964_RS00835 | TAGTAAGCGATTACAA |
| 6 | *rbsA* | A964_RS00840 | TAGTAAGCGATTACAA |
| 7 | *rbsC* | A964_RS00845 | TAGTAAGCGATTACAA |
| 8 | *rbsR* | A964_RS00850 | TAGTAAGCGATTACAA |
| 9 | *dhaK* | A964_RS08005 | TTGAAAACCCTAACAA |
| 10 | *eda2* | A964_RS03785 | AAGATAACGGTTACAT |
| 11 | *uxaC* | A964_RS03790 | AAGATAACGGTTACAT |
| 12 | *uxaA* | A964_RS03795 | AAGATAACGGTTACAT |
| 13 | *ptsG* | A964_RS08195 | AAGTAAGGGTTTACAA |
| 14 | *rok* | A964_RS08200 | AAGTAAGGGTTTACAA |
| 15 | *rgfB* | A964_RS08205 | AAGTAAGGGTTTACAA |
| 16 | *scrR* | A964_RS08210 | AAGTAAGGGTTTACAA |
| 17 | *scrB* | A964_RS08215 | AAGTAAGGGTTTACAA |
| 18 | *regR* | A964_RS08845 | ATGAAAGCGCTATAAT |
| 19 | *ulaD* | A964_RS08865 | ATGAAAGCGCTATAAT |
| 20 | *hylF* | A964_RS08870 | ATGAAAGCGCTATAAT |
| 21 | *hylE* | A964_RS08875 | ATGAAAGCGCTATAAT |
| 22 | *hylD* | A964_RS08880 | ATGAAAGCGCTATAAT |
| 23 | *scrA* | A964_RS08205 | AATGAAACGCTTTCAA |

**Additional file** **10** **The *cre* regions in the promoters of the downregulated genes in RNA-seq.**
